# Cytochemical and comparative transcriptome analyses elucidate the formation and ecological adaptation of three types of pollen coat in Zingiberaceae

**DOI:** 10.1186/s12870-022-03796-2

**Published:** 2022-08-20

**Authors:** Guo-Hui Lu, Jia-Ling Xu, Man-Xiang Zhong, Dong-Li Li, Min Chen, Ke-Ting Li, Ying-Qiang Wang

**Affiliations:** 1 Guangdong Provincial Key Laboratory of Biotechnology for Plant Development, School of Life Sciences, South China Normal University, Guangzhou, 510631 China; 2 Guangzhou Key Laboratory of Subtropical Biodiversity and Biomonitoring, School of Life Sciences, South China Normal University, Guangzhou, 510631 China

**Keywords:** Adaptive evolution, Evolutionary ecology, Ginger, Pollenkitt, Pollination, Positive selection, Reproductive ecology

## Abstract

**Background:**

The pollen ornate surface of flowering plants has long fascinated and puzzled evolutionary biologists for their variety. Each pollen grain is contained within a pollen wall consisting of intine and exine, over which the lipoid pollen coat lies. The cytology and molecular biology of the development of the intine and exine components of the pollen wall are relatively well characterised. However, little is known about the pollen coat, which confers species specificity. We demonstrate three types of pollen coat in Zingiberaceae, a mucilage-like pollen coat and a gum-like pollen coat, along with a pollen coat more typical of angiosperms. The morphological differences between the three types of pollen coat and the related molecular mechanisms of their formation were studied using an integrative approach of cytology, RNA-seq and positive selection analysis.

**Results:**

Contrary to the ‘typical’ pollen coat, in ginger species with a mucilage-like (*Caulokaempferia coenobialis*, Cco) or gum-like (*Hornstedtia hainanensis*, Hhn) pollen coat, anther locular fluid was still present at the bicellular pollen (BCP) stage of development. Nevertheless, there were marked differences between these species: there were much lower levels of anther locular fluid in Hhn at the BCP stage and it contained less polysaccharide, but more lipid, than the locular fluid of Cco. The set of specific highly-expressed (SHE) genes in Cco was enriched in the ‘polysaccharide metabolic process’ annotation term, while ‘fatty acid degradation’ and ‘metabolism of terpenoids and polyketides’ were significantly enriched in SHE-Hhn.

**Conclusions:**

Our cytological and comparative transcriptome analysis showed that different types of pollen coat depend on the residual amount and composition of anther locular fluid at the BCP stage. The genes involved in ‘polysaccharide metabolism’ and ‘transport’ in the development of a mucilage-like pollen coat and in ‘lipid metabolism’ and ‘transport’ in the development of a gum-like pollen coat probably evolved under positive selection in both cases. We suggest that the shift from a typical pollen coat to a gum-like or mucilage-like pollen coat in flowering plants is an adaptation to habitats with high humidity and scarcity of pollinators.

**Supplementary Information:**

The online version contains supplementary material available at 10.1186/s12870-022-03796-2.

## Background

Pollen grains are the microgametophytes of seed plants that produce the male gametes needed for sexual reproduction [1]. Each pollen grain is contained within a pollen wall consisting of intine and exine, over which the lipoid pollen coat lies [2, 3]. Pollen wall development is an elaborate process, which begins at the tetrad stage of microspores when the callose around microspores is degraded by callases secreted from the tapetum inside the anther [4]. At this stage, young microspores form primexine, composed mainly of cellulose, which acts as an elaborate template for the deposition of exine precursors [5, 6]. Sporopollenin is then synthesized in the tapetum and transported to form exine; after that, the intine, which is composed mainly of pectin, cellulose and hemicellulose, is generated by the microspore [7–9]. The exine of mature pollen grains is often covered by a pollen coat, which is formed at the final stages of pollen development [4, 10]. A genetic pathway, *SPL*/*NZZ*-*DYT1*-*TDF1*-*AMS*-*MS188*/*MYB80*-*MS1*, has been proposed to regulate pollen wall formation [11–14], and many genes that regulate the development of exine and intine have been reported [9, 15–17]. However, few studies have focused on the development of the pollen coat [18], a diversified element of the pollen wall.

Pollen coat refers to the adhesive material on the surface of pollen grains [19–21], such as pollenkitt, tryphine and elastoviscin, all of which are produced by secretion from and degeneration of the anther tapetum [22–24]. Pollenkitt, which is most common in dicots and monocots, is hydrophobic and is formed mainly from plastids (elaiosomes and/or spherosomes) of the anther tapetum [10, 23, 25]. Tryphine, which is found in Brassicaceae and is composed of a mixture of hydrophilic and hydrophobic substances, is generated by partial degeneration of the tapetum and its residual cytoplasmic contents [23, 26]. Elastoviscin, which is a more sticky substance found in Orchidaceae and Asclepiadaceae, is produced in the cytoplasm of tapetal cells without the participation of plastids [22, 27, 28]. In fact, both tryphine and elastoviscin are a special form of pollenkitt, and there is no great difference between these three types of pollen coat [22]. The most vital function of pollen coat is thought to be its role as an adhesive [10, 29–31]: it holds pollen grains in the anther until dispersal, enables secondary pollen presentation and maintains pollen grains in clumps prior to dispersal, and facilitates pollen adhesion to pollinators and stigmas. In addition, the pollen coat may also play an important role in pollinator attraction, recognition and compatibility at fertilization, and pollen hydration and germination, etc. [10]. Although the function and diversity of types of pollen coat have been cataloged in some detail, knowledge of the cytological morphology and related molecular mechanisms of pollen coat development is meager.

In general, the pollen coat is mainly composed of complex lipids, wax esters, carotenoids, flavonoids and proteins [10, 32]. Although the composition of the pollen coat varies, lipids always remain the primary constituent [33], and polysaccharides have only rarely been reported, e.g., in *Tylosema esculentum* [34] and three monocotyledon seagrasses, *Thalassia hemprichii*, *Halophila stipulacea* and *Thalassodendron ciliatumsome* [35]. It follows, therefore, that genes involved in long-chain fatty acids (LCFA) metabolism and transportation are essential for pollen coat formation. Four types of *ECERIFERUM* (*CER*) gene, including *CER1* [36], *CER3* [37], three *CER2-LIKE* genes [38] and *CER6* [39, 40], are thought to participate in LCFA metabolism during pollen coat formation; mutations in these genes result in defective pollen coat formation and male sterility. In addition, seven genes involved in pollen coat biosynthesis, *AtPKSA/B* [2, 41], *AtTKPR1/2* [42], *AtLAP3* [43] and *LACS 1/4* [44], which also participate in exine and cuticular wax biosynthesis, have been identified whose mutants display abnormal pollen coat and exine formation, together with male sterility. In addition, some genes, particularly the ABC transporters, which play essential roles in tapetum development, may also influence the transport of substances involved in pollen coat formation, since the tapetum plays a vital role in the formation and transportation of pollen coat precursors. For example, *ABCG9* and *ABCG31* are highly expressed in the tapetum and are involved in pollen coat deposition [45]. Loss of *ABCG1* and *ABCG16* function causes abnormalities in the cellular structures (e.g., tapetosomes and elaioplasts, which play a role in the transport of pollen coat components) and metabolism of tapetal cells, and particularly affects processes related to pollen coat materials [46]. However, this information on all of the above genes is primarily derived from molecular genetics and biochemical or cellular biology studies of the respective male-sterile mutants, rather than from specific studies on the development and formation of pollen coat. The chemical nature of pollen coats and the pathways by which they are biosynthesized and transferred to the pollen surface are poorly understood [24, 47].

**Fig. 1:**
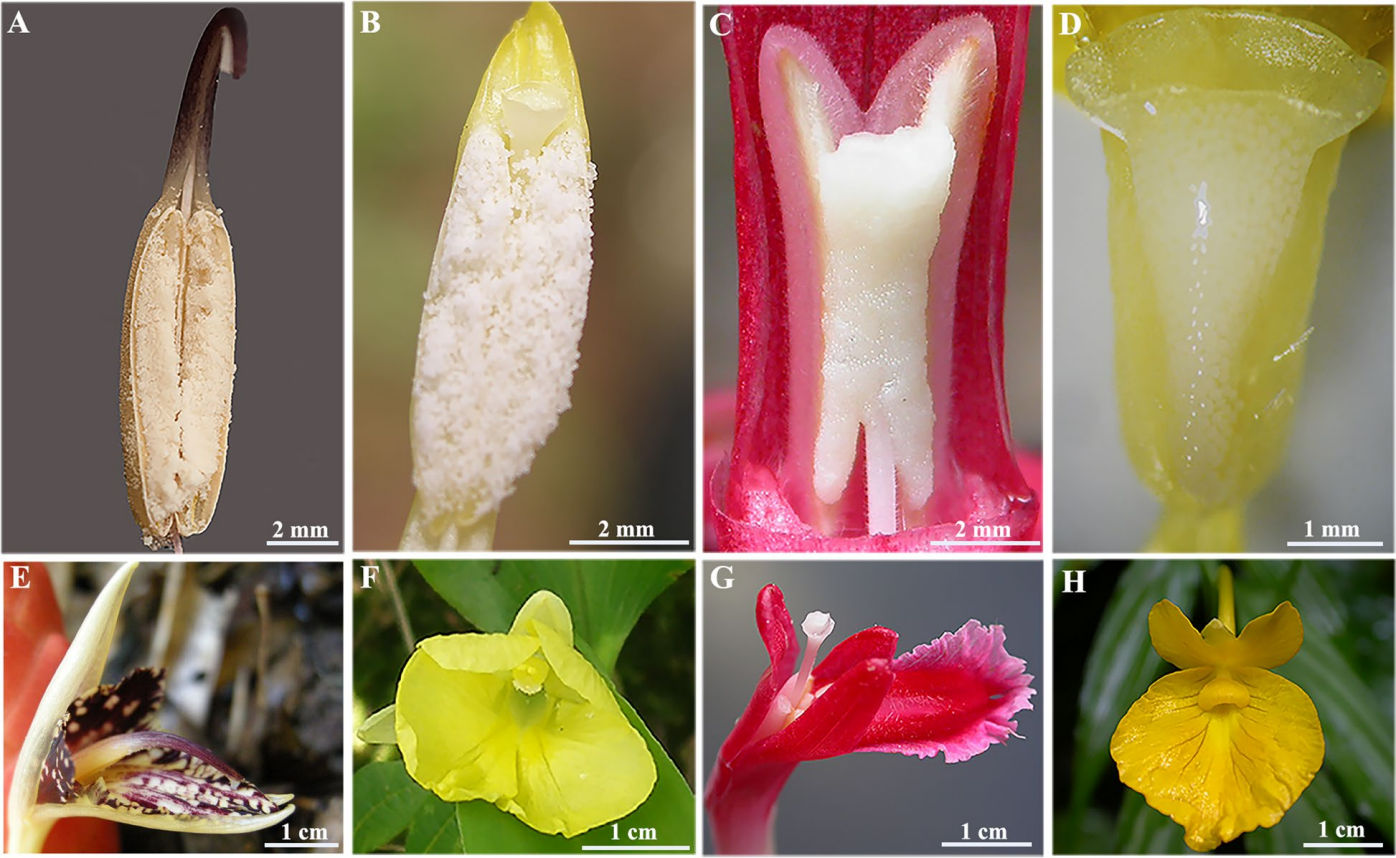
Flowers and anthers of *Zingiber nudicarpum*. (**A**/**E**), *Pyrgophyllum yunnanense*. (**B**/**F**), *Hornstedtia hainanensis*. (**C**/**G**), *Caulokaempferia coenobialis* (**D**/**H**). **A** Anther of *Z. nudicarpum*, pollen grains are distributed singly. **B** Anther of *P. yunnanense*, pollen grains are distributed singly. **C** Anther of *H. hainanensis*, pollen grains are enclosed in a gum-like pollen coat. **D** Anther of *C. coenobialis*, pollen grains are covered in a mucilage-like pollen coat. Scale bars: **A**, **B**, **C**: 2 mm; **D**: 1 mm; **E**, **F**, **G**, **H**: 1 cm)

The Zingiberaceae, consisting of approximately 50 genera and 1,300 species [48], is a naturally monophyletic group [49, 50]. However, members of this family display a broad range of pollination and breeding systems, including obligate xenogamy, facultative xenogamy and autogamy; they are also pollinated by many different animals, such as bees, sunbirds and other vertebrates, beetles and moths [51–57]. During more than a decade of fieldwork in tropical and subtropical China (since 2002), we have found that there are three types of pollen coat in Zingiberaceae plants. These are 1) the pollen coat typical of the majority of ginger species, e.g., *Zingiber nudicarpum* D. Fang (Fig. 1A) and *Pyrgophyllum yunnanense* (Gagnepain) T. L. Wu & Z. Y. Chen (Fig. 1B), which is similar to that of most angiosperms, such as rice and *Arabidopsis thaliana*, where the pollen grains have a small amount of pollen coat and are distributed singly; 2) the gum-like pollen coat found in species of *Curcuma* and *Hornstedtia*, e.g., *C. kwangsiensis* and *H. hainanensis* T. L. Wu & S. J. Chen (Fig. 1C), in which pollen grains are covered in a gum-like substance; and 3) the mucilage-like pollen coat found in *Caulokaempferia coenobialis* (Hance) K. Larsen (Fig. 1D), in which pollen grains are coated in mucilage [54, 55, 58]. Therefore, the Zingiberaceae represent an ideal system in which to study the formation and development of the pollen coat and the evolutionary significance of these processes. With the rapid development of next-generation sequencing (NGS), RNA-seq has become more efficient and less expensive and is increasingly being used to reveal candidate genes with a potential role in adaptation to the environment and in the organ development of non-model plants [59–64].

In this paper, four ginger species with three types of pollen coat, i.e., *C. coenobialis* (mucilage-like pollen coat), *H. hainanensis* (gum-like pollen coat) and two species with what we will term a ‘typical’ pollen coat (*P. yunnanense, Z. nudicarpum*), were chosen to study the cytological morphology and related molecular mechanisms of the development of the various pollen coats. We adopted an integrative approach, including cytology, RNA-seq and positive selection analysis (using the PAML), to elucidate the differences in pollen coat development between the three types. The aims were to explore: 1) differences in cytological morphology and chemical nature of the three types of pollen coat; 2) which differentially expressed genes might be associated with the formation of each pollen coat type and to identify candidate genes involved in pollen coat development; 3) the ecological adaptive significance of the different types of pollen coat. These results should serve as a foundation for understanding the development, evolution and adaptation of pollen coat in response to pollination strategies and habitats in Zingiberaceae.

## Results

### Cytological analysis of different pollen development stages

**Fig. 2:**
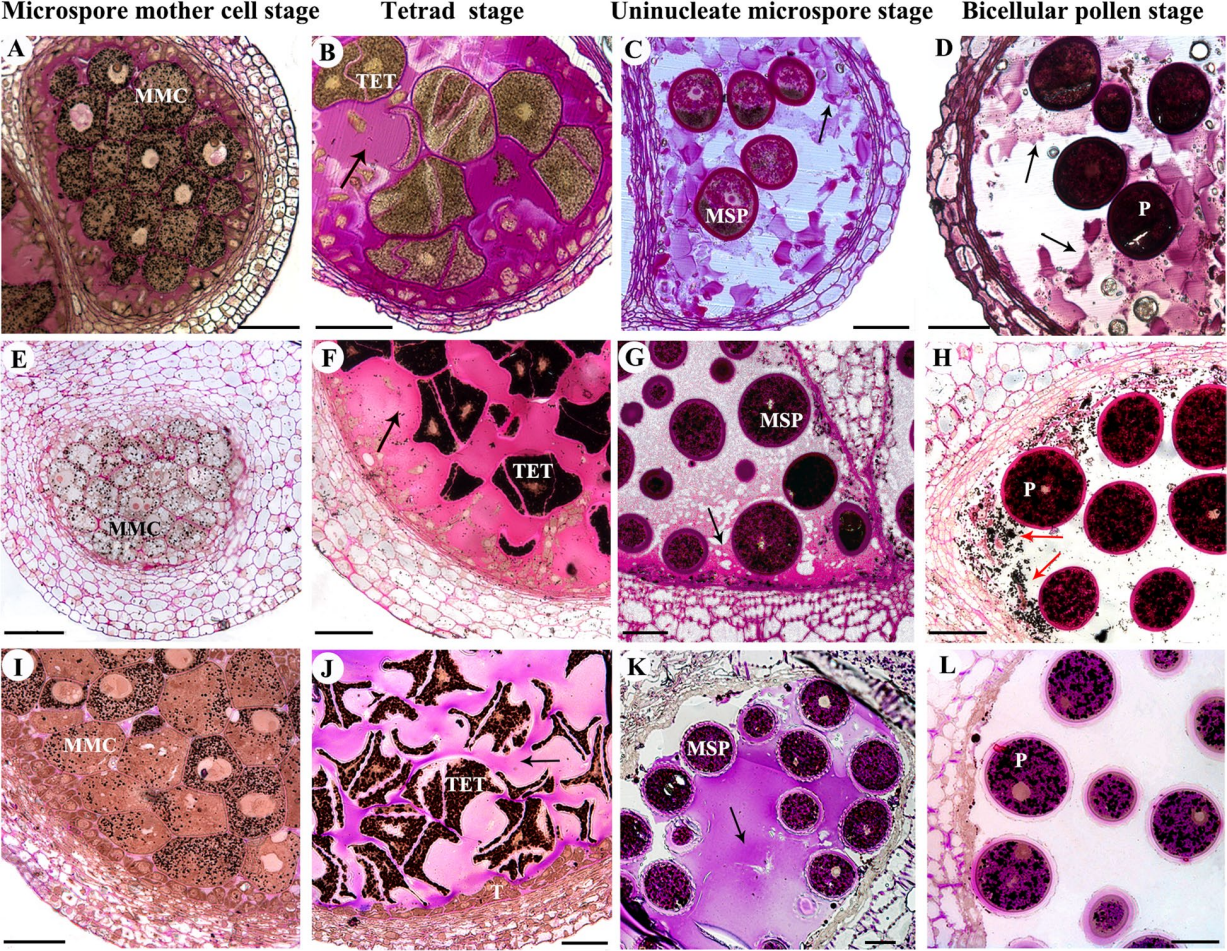
Transverse anther sections of three ginger species at different developmental stages stained with Periodic acid—Schiff (PAS, a staining method used to detect polysaccharides) and Sudan Black B (a staining method used to detect lipid), showing the distribution of polysaccharides and lipids in different anther tissues. Polysaccharides stained red, lipid stained black dots. **A** to **D**, *Caulokaempferia coenobialis.* **E** to **H**, *Hornstedtia hainanensis*. **I** to **L**, *Zingiber nudicarpum.* MMC, microspore mother cell; MSP, microspores; P, pollen grain; T, tapetum; TET, tetrad; the arrow shows the liquid in the locule. Scale bars, 50 μm

Our cytological observations showed that the anthers of all four ginger species were tetrasporangiate. In transverse sections of the MMC (Microspore mother cell) stage (Fig. 2A, E, I; Fig. S1A), the microspore mother cells of all four ginger species were oval or angular in shape and arranged closely. There was no locular fluid in the microsporangia.

At the TET (Tetrad) stage (Fig. 2B, F, J; Fig. S1B), the anther locules of the four ginger species enlarged and the locular fluid began to form. The tetrads of haploid microspores were surrounded by a callose envelope and the locular fluid filled the anther locules. In addition, large amounts of red PAS-positive substances accumulated in the wall of all tetrads and in the locular fluid of all four ginger species, indicating that both contain callose and polysaccharides. To the uninucleate microspore stage, the callose was degraded and there were large amounts of locular fluid, which contains red PAS-positive substances in the locules and surrounded the uninucleate microspore (Fig. 2C, G, K; Fig. S1C).

At the late BCP (Bicellular pollen) stage (Fig. 2D, H, L; Fig. S1D), the anther locules enlarged further, resulting in a large amount of space between bicellular pollen grains. In the locules of Cco (*Caulokaempferia coenobialis*), the amount of locular fluid, which contains high levels of polysaccharide but low levels of lipid, remained constant in the locules and surrounded the bicellular pollen grains. Compared with Cco, in the locules of Hhn (*Hornstedtia hainanensis*) the amount of locular fluid, together with its polysaccharide content, decreased markedly at the late BCP stage, while lipids, Sudan Black B positive substances, were clearly detected and accumulated to higher levels. In contrast, the locular fluid disappeared in Pyn (*Pyrgophyllum yunnanense*) and Znu (*Zingiber nudicarpum*) at the BCP stage.

### Gene annotation and functional classification

**Fig. 3:**
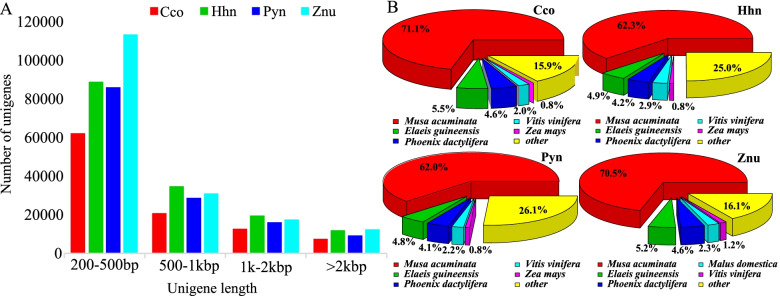
Length distributions of unigenes and species distribution based on annotation by the NR database with BLAST for four ginger species, *Caulokaempferia coenobialis* (Cco), *Hornstedtia hainanensis* (Hhn), *Pyrgophyllum yunnanense* (Pyn), and *Zingiber nudicarpum* (Znu). **A** length distributions of unigenes; **B** species distribution of the top Blast hits

A total of 103500–174594 unigenes were generated from the four ginger species by Illumina sequencing with an average length for each species in the range of 682–763 bp. The N50 of the four species ranged from 1098 to 1213 bp. Detailed de novo assembly results are summarized in Table S1. Unigenes with lengths between 200–500 bp were overrepresented, making up at least 57% of the total number of unigenes (60.16% for Cco, 57.21% for Hhn, 61.34% for Pyn, and 64.98% for Znu). Fewer than 8% (7.30%, 7.76%, 6.61% and 7.16%, respectively) of the unigenes were longer than 2000 bp (Fig. 3A).

After sequence contig assembly, unigenes were annotated by comparison with seven functional databases: NCBI NR and NT, KEGG, SwissProt, PFAM, GO and KOG. Proteins in the NR database and the SwissProt database gave a match with 26.53 to 39.37% and 22.70-32.48% of the unigenes, respectively. Among these unigenes, 4102–9979 unigenes (3.17-5.71%) were annotated by all seven databases, and 40660–96876 unigenes (38.56-55.48%) were successfully annotated by at least one database (Table S2). Concerning species similarity, the highest proportion of matched sequences of Cco in the NR database derived from *Musa acuminata* (71.1%, 25681 unigenes). Other species matched at no more than 5.5%, including *Elaeis guineensis* (Arecaceae) with 5.5% and *Phoenix dactylifera* (Arecaceae) with 4.6% (Fig. 3B). The other three ginger species, Hhn, Pyn, and Znu, gave similar results (Fig. 3B).

To understand the potential function of the assembled unigenes, the KOG (eukaryotic ortholog groups) functional annotation system was used. KOG-annotated putative proteins were classified into 25 groups, among which the cluster for ‘posttranslational modification, protein turnover, chaperones’ was the largest group (13.55%, 12.48%, 13.80%, and 14.23% of the matched unigenes in Cco, Hhn, Pyn and Znu, respectively), followed by ‘general function prediction only’ in Cco and Hhn, but by ‘translation, ribosomal structure, and biogenesis’ in Pyn and Znu. The smallest group was ‘cell motility’ in all four ginger species. Detailed information on KOG annotations of putative proteins for the four species is given in Table S3.

Gene ontology assignments were used to classify the functions of predicted genes, resulting in 22.56-29.02% unigenes being assigned to at least one GO term (Table S2). The BLASTable (*E*-value < 1e-6) unigenes were divided into three GO categories: biological process, cellular component and molecular function. The constitution of the three GO categories within the four ginger anther transcriptome profiles was basically consistent (Fig. S2). In addition, the assembled unigenes were annotated against the KEGG database (E-value < 1e-10) to identify biological pathways involved in anther development in the four ginger species. A total of 9.28-15.68% unigenes were mapped in the KEGG database (Table S2). The top three KEGG classifications were ‘carbohydrate metabolism’, ‘translation’ and ‘folding, sorting and degradation’. This profile was very similar for all four anther transcriptomes (Table S4).

### Identification of transcripts differentially expressed across the four ginger species

**Fig. 4:**
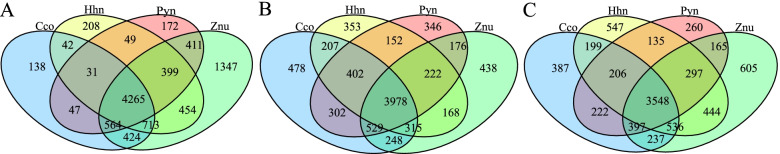
Venn diagram of the highly expressed unigenes at the three different pollen developmental stages of four ginger species, *Caulokaempferia coenobialis* (Cco), *Hornstedtia hainanensis* (Hhn), *Pyrgophyllum yunnanense* (Pyn), and *Zingiber nudicarpum* (Znu). The overlaps represent unigenes simultaneously highly expressed in more than one species. **A** microspore mother cell stage; **B** tetrad stage; **C** bicellular pollen stage

The number of expressed unigenes of the four ginger species at the three developmental stages were 82046–137234 (MMC), 76438–139559 (TET) and 70013–152028 (BCP). The FPKM values varied widely, from 0.3 to 400286 (a FPKM value greater than 0.3 is regarded as confirming the unigene is expressed), of which the number of highly expressed unigenes in the four ginger species that were detected at the MMC, TET and BCP stages were 19098–34310, 19111–34904 and 17504–38007, respectively. Further GO and KEGG functional analyses focused on these highly expressed genes (Fig. 4).

**Fig. 5:**
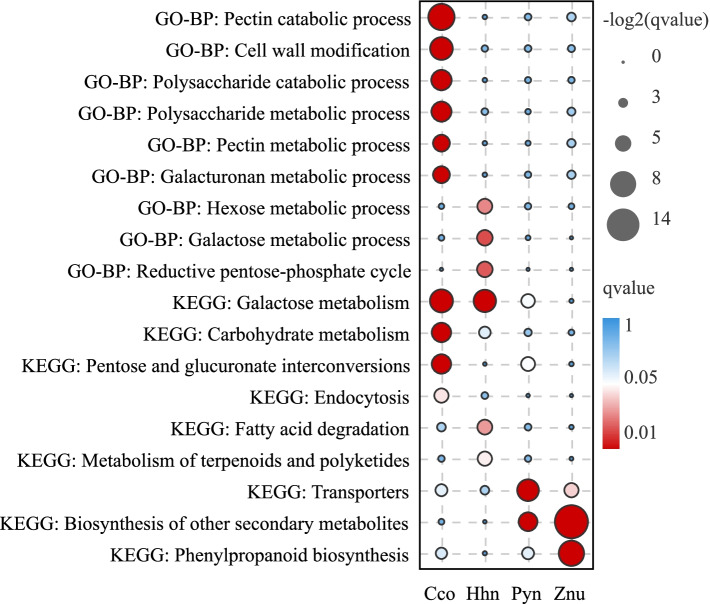
Enriched GO Biological Process (GO-BP) terms and KEGG pathways of specific highly expressed genes at bicellular pollen stage in four ginger species, *Caulokaempferia coenobialis* (Cco), *Hornstedtia hainanensis* (Hhn), *Pyrgophyllum yunnanense* (Pyn) and *Zingiber nudicarpum* (Znu)

The cytology results revealed that the main events affecting the type of pollen coat occurred at the BCP stage. Accordingly, the specific highly expressed genes (SHE) at the BCP stage of all four ginger species (Fig. 4C) were selected to understand the genetic basis for the different types of pollen coat: SHE-Cco refers to unigenes that were only highly expressed in Cco compared to Pyn and Znu; SHE-Hhn refers to unigenes that were only highly expressed in Hhn compared to Pyn and Znu; SHE-Pyn refers to unigenes that were only highly expressed in Pyn compared to Cco and Hhn; while SHE-Znu refers to unigenes that were only highly expressed in Znu compared to Cco and Znu. Functional analysis of these SHE unigene sets based on GO and KEGG annotations (Fig. 5, Table S5) revealed that SHE-Cco unigenes at the BCP stage were significantly enriched in ‘pectin catabolic process’, ‘polysaccharide catabolic process’ and ‘galacturonan metabolic process’, etc. according to the GO database. These unigenes were also significantly enriched in ‘galactose metabolism’, ‘carbohydrate metabolism’, and ‘endocytosis’, etc. according to the KEGG analysis. SHE-Hhn unigenes were found to be significantly enriched in ‘hexose metabolic process’, ‘reductive pentose-phosphate cycle’, and ‘cellular amino acid metabolic process’, etc. according to the GO analysis. Categorization of SHE-Hhn sets into KEGG functional groups showed significant enrichment of unigenes in ‘galactose metabolism’, ‘fatty acid degradation’ and ‘metabolism of terpenoids and polyketides’, etc. (Fig. 5, Table S5). SHE-Pyn unigenes were involved in ‘protein families: signaling and cellular processes’, ‘transporters’, and ‘biosynthesis of other secondary metabolites’, etc. according to the KEGG analysis (Fig. 5, Table S5). For the SHE-Znu set, KEGG analysis showed a high degree of enrichment for ‘biosynthesis of other secondary metabolites’, ‘phenylpropanoid biosynthesis’, and ‘carbohydrate metabolism’, along with ‘transporters’ (Fig. 5, Table S5). SHE-Pyn and SHE-Znu unigenes were not significantly enriched in any GO terms associated with organic metabolism except for ‘secondary metabolic process’ in SHE-Znu. Nevertheless, the unigenes of both sets were enriched in ‘transporters’ based on KEGG analysis.

### Identification of putative candidate genes related to pollen coat development

Using OrthoFinder, a total of 2605 putative orthologs were identified by comparing the four ginger transcript sets. These orthologs, together with orthologs in *A. thaliana*, in which the genes of pollen wall development have been well studied, were used in the subsequent evolutionary analysis.

To estimate lineage-specific evolutionary rates for the Cco and Hhn branches compared to the other two branches, we used branch models that allow the ω ratio to vary among branches across the phylogeny tree ((Znu, Cco), Pyn, Hhn), which was generated by the iqtree module in OrthoFinder. The likelihood ratio tests showed that 18 unigenes (10 orthologs in *A. thaliana*) in the Cco lineage and 23 unigenes (12 orthologs in *A. thaliana*) in the Hhn lineage underwent positive selection pressure (Table S6). In the Cco lineage, the *A. thaliana* ortholog c74617_g1 (AT3G53510 / *ABCG20*) is required to synthesize suberin and an intact pollen wall, while c73851_g1 (AT3G02850 / *SKOR*), c74617_g1 (AT3G53510 / *ABCG20*) and c16484_g2 (AT1G23090 / *AST9*) are associated with transport functions. In the Hhn branch, c39068_g1 (AT1G12500), c45170_g2 (AT3G02850 / *SKOR*), c19830_g2 (AT4G02700 */ SULTR3;2*) and c55055_g1 (AT1G64780 / *AMT1;2*) have material transport functions, c120589_g1 (AT4G00360 / *ATT1*) is related to cutin biosynthesis [65], and c15019_g1 (AT5G06090 / GPAT7) is involved in CDP-diacylglycerol and suberin biosynthesis [66].

**Fig. 6:**
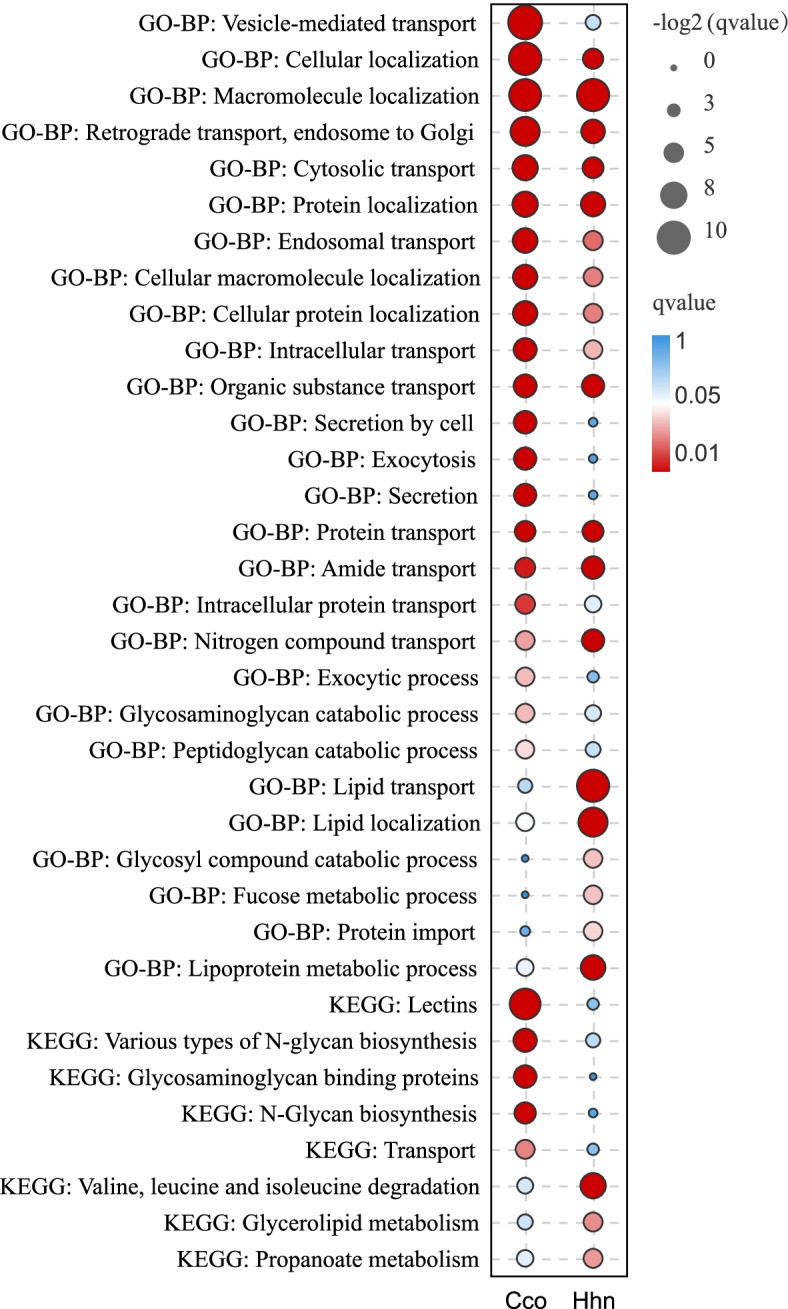
Enriched GO Biological Process (GO-BP) terms and KEGG pathways in positively selected genes of two ginger species branches, *Caulokaempferia coenobialis* (Cco) and *Hornstedtia hainanensis* (Hhn)

To detect positive selection of a few codons in a specific lineage, we used the optimized branch-site model [67]. In total, we identified 524 (230 orthologs in *A. thaliana*) and 604 (279 orthologs in *A. thaliana*) PSGs in the Cco and Hhn lineages, respectively (Table S7). Functional enrichment (Fig. 6, Table S8) analysis showed that, according to the KEGG database, the PSGs identified in the Cco lineage were significantly enriched for genes involved in ‘lectins’, ‘various types of N-glycan biosynthesis’, ‘glycosaminoglycan binding proteins’ and ‘transport’. In the Hhn lineage, the PSGs were significantly enriched in **‘**valine, leucine and isoleucine degradation’, ‘glycerolipid metabolism’ and ‘propanoate metabolism’ based on KEGG analysis. PSGs in Cco and Hhn were both significantly enriched in transport and localization according to the GO database, with terms such as ‘cytosolic transport’, ‘intracellular transport’, ‘organic substance transport’, ‘retrograde transport, endosome to Golgi’, ‘cellular macromolecule localization’ and ‘cellular protein localization’. Terms specific to a single lineage included ‘vesicle-mediated transport’, ‘exocytic process’ and ‘secretion by cell’ for Cco, and ‘lipid transport’ and ‘lipid localization’ for Hhn.

Based on the pollen coat material and previously reported genes associated with pollen wall development, we identified 59 and 72 candidate PSGs that may be involved in pollen coat development in Cco and Hhn mainly associated with lipid metabolism, carbohydrate metabolism and transport, respectively (Table S9).

## Discussion

### Different types of pollen coat in ginger species depend on the residual amount and composition of anther locular fluid at the bicellular pollen stage

In angiosperms, pollen develops in a closed loculus surrounded by the tapetum. During pollen development, the pollen grains are immersed in locular fluid (nutritive fluid) secreted by the tapetum that conveys nutrients from the sporophyte to the developing pollen [68, 69]. The locular fluid is thought to begin to form at the beginning of the dyad-tetrad stage, with the composition of the locular fluid changing during different stages of pollen development [23, 69]; we confirmed this in our cytological observations of the four ginger species (Fig. 2). Previous studies showed that the locular fluid contains mainly polysaccharides, pectins and proteins at an early stage of pollen development [69–71], then mainly lipid from pollen mitosis to anthesis [69], after which it disappears when the pollen is almost ripe by reabsorption, evaporation, or both, to enable pollen dispersal [72, 73]. Eventually, in almost all angiosperms, the tapetum degenerates and produces viscous substances (i.e., pollen coat) that cause the pollen to clump together [73]. Similarly, our cytological analysis with PAS and Sudan Black B staining showed that the anther loculus of all four ginger species at the TET stage were filled with fluid containing large amounts of polysaccharide and small amounts of lipid (Fig. 2). As in most angiosperms, including for example *Lilium* [69] and *Arabidopsis thaliana* [32], the locular liquid disappeared during the last stage of pollen development in Pyn and Znu (‘typical’ pollen coat) (Fig. 2), probably by reabsorption, to enable pollen dispersal.

However, unlike in previous studies, there were still a significant amount of locular fluid containing large amounts of polysaccharide as well as some lipid at the late BCP stage in Cco (mucilage-like pollen coat), and small amounts of locular fluid in Hhn (gum-like pollen coat) with much lipid as well as some polysaccharide (Fig. 2). Thus, the development of both the mucilage-like pollen coat (e.g., Cco) and the gum-like pollen coat (e.g., Hhn) is different from that of the ‘typical’ pollen coat of most angiosperms (such as rice, *A. thaliana,* Pyn and Znu). In other words, in contrast to most angiosperms, the locular fluid in species with mucilage-like and gum-like pollen coats has been maintained from pollen mitosis to anthesis. Functional annotation of SHE unigene sets also showed that ‘transporters’ were enriched at the BCP stage in species with a ‘typical’ pollen coat (Pyn, Znu), but not in species with a gum-like pollen coat (Hhn) or a mucilage-like pollen coat (Cco). However, the amount and composition of the locular fluid in these latter two types of pollen coat are different. Compared with the mucilage-like pollen coat (Cco), the amount of locular fluid and polysaccharide in gum-like pollen coat (Hhn) decreased markedly at the late BCP stage, while lipid accumulated (Fig. 2). This was also consistent with the annotation results of SHE-Cco and SHE-Hhn unigenes. SHE-Cco unigenes were enriched in the terms ‘polysaccharide metabolic process’ and ‘pectin metabolic process’, while in SHE-Hhn unigenes ‘fatty acid degradation’ and ‘metabolism of terpenoids and polyketides’ were significantly enriched (Fig. 5).

Here, we suggest that the different types of pollen coat depend on the quantity and composition of anther locular fluid at the BCP stage during pollen development. When the locular fluid has disappeared by the later stages of pollen development, a ‘typical’ pollen coat will be formed. However, when the amount of locular fluid remains constant from pollen mitosis to anthesis, and it contains a large amount of polysaccharide and small amounts of lipid, a mucilage-like pollen coat will be formed. Finally, when the amount of locular fluid and the amount of polysaccharide it contains both decrease markedly at the late BCP stage, but the lipid content accumulates, a gum-like pollen will be formed. Although the mucilage-like (Cco), gum-like (Hhn) and typical (Pyn, Znu) pollen coats differ in viscosity and composition, and in the way the tapetum degenerates during their formation, they are homologous because they are all derived from the final degradation of the anther tapetum [23, 24].

### Genes potentially involved in mucilage-like and gum-like pollen coat development in Zingiberaceae

Pollen development has been widely studied in *Arabidopsis* and rice, and the results obtained suggest a conserved pathway [9, 15–17, 74, 75]. A genetic pathway, *SPL*/*NZZ*-*DYT1*-*TDF1*-*AMS*-*MS188*/*MYB80*-*MS1*, has been proposed to regulate pollen wall formation [11–14]. To date, many genes involved in tapetum or pollen wall development have been identified. For example, *TDF1* (AT3G28470) plays a vital role in the differentiation and function of tapetum [76] and is highly expressed at the MMC stage. Our analysis of the homologs of *TDF1* showed the same expression profiles in the four ginger species (Table S10). *AtSUP* (AT5G52560) is suggested to be involved in intine development and is putatively highly expressed at the BCP stage [77]. The homologs of *AtSUP* in the four ginger species were also highly expressed at the BCP stage (Table S10). Therefore, pollen wall development in the four ginger species seems likely to involve the same conserved pathway as *Arabidopsis* and rice.

However, the functions of the homologous genes involved in pollen development may have diversified between species during plant evolution [15], due to the morphological differences of pollen wall structure. Thus, the pollen wall of Zingiberaceae, which consists of a significantly reduced, thin (membrane-like) exine and a very thick (2–4 layers) and elaborate intine structure [78, 79], differs from that of *Arabidopsis* and rice. In this case, we would expect the expression pattern of their homologous genes to be different from that of *Arabidopsis* and there are data to support this. For example, *LAP3* (AT3G59530), *LAP5* (AT4G34850), *LAP6* (AT1G02050) and *ABCG26* (AT3G13220) are specifically expressed in *Arabidopsis* during the period of exine synthesis and are essential for exine production [2, 43, 80]. However, their homologs in the four ginger species are not expressed at all during this period. In addition, *TKPR1* (AT4G35420), which in *Arabidopsis* is involved in a sporopollenin monomer biosynthesis pathway and is also essential for exine production [42], shows high expression at the MMC stage and the TET stage. In contrast, the homologs in the ginger species studied, with the exception of Hhn, showed relatively low expression (0.81–14.44) at the same developmental stages (Table S10). Moreover, peak expression of the *TKPR1* homolog (283.54) in Hhn was delayed to the BCP stage, suggesting that sporopollenin biosynthesis is blocked in Hhn and that some intermediates are retained, perhaps being involved in synthesis of the gum-like pollen coat. Further experimental studies of the function of these genes are needed, but the above could explain why Zingiberaceae pollen has a significantly reduced thin exine.

Functional enrichment analysis showed that the PSGs likely to be involved in pollen coat development in Cco and Hhn are significantly enriched in transport and localization annotation terms. This seems logical since, during pollen development, the nutritive fluid (locular fluid) is secreted by the tapetum and transported to the developing pollen [68, 69], then is finally reabsorbed and/or lost by evaporation as pollen matures [73]. These processes may require the involvement of many enzymes primarily involved in transport and localization. However, the locular liquid in Cco and Hhn does not disappear at anthesis, in contrast to the situation in most angiosperms that form the ‘typical’ pollen, e.g. Pyn and Znu. Thus, the genes involved in transport and localization in mucilage-like (i.e., Cco) and gum-like (i.e., Hhn) pollen coat development have probably evolved under positive selection pressure. Furthermore, our results suggest that the PSGs significantly enriched in the terms ‘various types of N-glycan biosynthesis’, ‘glycosaminoglycan binding proteins’, ‘vesicle-mediated transport’, ‘exocytic process’ and ‘secretion by cell’ are specific to Cco, while the PSGs significantly enriched in the terms ‘glycerolipid metabolism’, ‘lipid transport’, ‘lipid localization’ are specific to Hhn (Fig. 6). This result was consistent with both the cytological analysis with PAS and Sudan Black B staining, and the enrichment analysis in SHE-Cco and SHE-Hhn (Fig. 2). The above suggest that the genes involved in ‘polysaccharide metabolism’ and ‘transport’ in the development of a mucilage-like pollen coat (Cco) and in ‘lipid metabolism’ and ‘transport’ in the development of a gum-like pollen coat (Hhn) have likely evolved under positive selection. In addition, Cco PSGs were significantly enriched in the terms ‘vesicle-mediated transport’, ‘exocytic process’, and ‘secretion by cell’, which may be related to the fact that the amount of locular fluid remained constant in the locules during pollen development. Further experimental studies of functional genes are needed to clarify these issues.

As far as we know, only two of the 59 Cco and 72 Hhn candidate PSGs have been reported to be involved in pollen coat formation. The *Arabidopsis* homologs of these genes are AT1G68530 (At*CER6*) and AT2G38110 (*GPAT6*). The first of these is involved in synthesizing very-long-chain fatty acids, which are components of the pollen coat; indeed, the pollen of *cer6* mutant plants has a reduced pollen coat with few lipid droplets [39, 81]. GPAT6 is vital for ER assembly in the tapetum and the pollen coat of a *gpat6* mutant is inadequately loaded [82]. Apart from these two examples, the functions of the other candidate PSGs implicated in pollen coat development have not yet been elucidated. These genes, which display signatures of positive selection, will serve as a baseline for further investigating the characteristics of the pollen coat at both the molecular and phenotypic levels. Future investigations involving both experimental and functional genomics could provide insights into plant adaptation to the environment.

### The mucilage-like and gum-like pollen coats in Zingiberaceae are examples of remarkable ecological adaptation to habitats with high humidity and scarcity of pollinators

Angiosperm pollen grains may be transported to the stigma by various biotic and abiotic mechanisms in monads or in groups [24], for which pollen structures are adapted [83, 84]. The main function of the pollen coat is to stick single pollen grains together to adhere to a pollinator’s body [10], enhancing pollination efficiency. In most angiosperms, the pollen usually has only a limited amount of pollen coat, which is not sufficient to fulfill the adhesive function when the pollen must adhere to smooth surfaces on pollinators [85], such as the smooth beak and mandible of birds and Lepidoptera, respectively. This can be a problem for plants that grow in habitats that lack pollinators, such as forest habitats with high humidity and alpine habitats. In these cases, a large amount of pollen coat becomes important [86], because it provides a highly viscous fluid that adheres to the pollinator to improve pollination efficiency [87]. This is the case for Cco and Hhn in our study of Zingiberaceae, a naturally monophyletic group found in the tropics and subtropics [48–50]. Bee pollination is thought to be the ancestral pollination route of Zingiberaceae, but this has subsequently undergone many independent transitions from bee pollination to vertebrate, sunbird, beetle and moth pollination [88]. Hhn and Cco both grow in high humidity habitats near streams in subtropical forest valleys. Our field observations showed that Hhn and *Hornstedtia scottiana* share the same flower design (Fig. 1G) and gum-like pollen coat wrapping of their pollen grains, and both exhibit a delayed self-pollination mechanism involving elongation of the corolla tube [51] when cross-pollination does not occur due to the lack of pollinators. However, *H. scottiana* is mainly pollinated by the honeyeater (a sunbird) in tropical stream banks [51], suggesting that a gum-like pollen coat may be produced as a result of the transition from bee pollination to sunbird pollination. Nevertheless, we observed no pollinators of Hhn in five years of fieldwork. For Cco, a mucilage-like pollen coat plays a vital role in the self-pollination process, in which a film of pollen is transported from the anther (pollen sacs) by a liquefied pollen coat that slides sideways along the flower’s style and into the individual’s own stigma [54]. Although Cco is self-pollinating, it retains characteristics of a butterfly-pollinated flower (Fig. 1H) [58]. This suggests that a mucilage-like pollen coat may be a result of a transition from bee pollination to moth pollination, and then to self-pollination for reproductive security.

Here we suggest that shifting from a ‘typical’ pollen coat to a gum-like or mucilage-like pollen coat in Zingiberaceae is an adaptation to habitats with high humidity and scarcity of pollinators. Similar, but evolutionarily independent shifts have probably occurred in other angiosperm groups, such as *Souroubea guianensis* in the family Marcgraviaceae [89], *Symphonia globulifera* in the family Clusiaceae [90] and *Chamelaucium uncinatum* in the family Myrtaceae [91]. In habitats with high humidity and a lack of pollinators, a large amount of sticky pollen coat can hold pollen grains in the anther, keeping the grains together during pollination, and protecting them from being washed away before pollinators, especially ‘low-grooming’ pollinators (e.g., birds and Lepidoptera), can visit [90, 92]. In addition, a large amount of sticky pollen coat is conducive to more pollen adhering to the smooth surfaces of birds or Lepidoptera, thereby improving pollination efficiency.

## Conclusions

This paper is the first genomic resource for pollen development in Zingiberaceae and provides novel insights into the cytological morphology and molecular mechanisms of different types of pollen coat development. Our cytological and comparative transcriptome analysis showed that different types of pollen coat depend on the residual amount and composition of anther locular fluid at the BCP stage. Contrary to most angiosperms, which have a ‘typical’ pollen coat, there were still large amounts of locular fluid containing large amounts of polysaccharide but few lipids at the late BCP stage in Cco (mucilage-like pollen coat), and small amounts of locular fluid with a large amount of lipid in Hhn (gum-like pollen coat). The genes involved in ‘polysaccharide metabolism’ and ‘transport’ in the development of a mucilage-like pollen coat (Cco) and in ‘lipid metabolism’ and ‘transport’ in the development of a gum-like pollen coat (Hhn) probably evolved under positive selection in both cases. We suggest that the shift from a typical pollen coat to a gum-like or mucilage-like pollen coat in Zingiberaceae represents an adaptation to habitats with high humidity and scarcity of pollinators.

## Materials and methods

### Species, sample preparation and cytological analysis of different pollen development stages

*Caulokaempferia coenobialis* (Hance) K. Larsen (Cco) is a deciduous perennial herb up to 50 cm in height that is endemic in south China, where it grows on humid cliffs, usually along streams in monsoon forests. The plant flowers from May to August (Fig. 1H), and the pollen grains are suspended in the mucilage-like pollen coat, which helps the pollen slide towards the stigma to achieve self-pollination [54, 55, 58]. *Hornstedtia hainanensis* T. L. Wu & S. J. Chen (Hhn) is a large evergreen rhizomatous herb up to 2.5 m in height with short, lateral, scarlet inflorescences that can be half-embedded in the ground, which is also endemic in south China, where it mainly grows in valleys or on hillsides alongside streams in humid monsoon forests. The plant flowers from March to May (Fig. 1G), and the pollen grains are held by a gum-like pollen coat in the anthers until the corolla tube elongates to achieve delayed self-pollination, similarly to *Hornstedtia scottiana* [51]. *Pyrgophyllum yunnanense* (Gagnepain) T. L. Wu & Z. Y. Chen (Pyn) is a deciduous perennial herb up to 55 cm in height with short rhizomes and tuberous roots, occurring in open forests or habitats with scattered trees and shrubs at an altitude of 1300–2800 m; it is endemic to southwest China. The plant flowers from July to September (Fig. 1F) and is characterised by various out-crossing pollination syndromes; its flowers are seldom visited by insects and thus are mostly self-pollinated [53, 55]. *Zingiber nudicarpum* D. Fang (Znu) is an evergreen perennial herb up to 2.5 m in height that is mainly distributed in south China, Vietnam and Thailand, where it often grows on the margins of mountains in broad-leaved evergreen forest. The plant flowers from May to July (Fig. 1E) and is usually cross-pollinated by a parasitic bee [93]. Although the habitats and pollination mechanisms of Pyn and Znu are different, their pollen grains both contain a limited amount of pollen coat (i.e. a ‘typical’ pollen coat) are distributed singly. The herbarium vouchers of *C. coenobialis* (WYQ-HHDBJ-5), *H. hainanensis* (WYQ-06–1), *P. yunnanense* (LGH-BYJ-2) and *Z. nudicarpum* (WYQ-14–24) were deposited in the Herbarium of School of Life Science, South China Normal University (SN). The species was identifed by professor Ying-Qiang Wang from School of Life Sciences, South China Normal University. The field work permits were obtained from the Baichong Provincial Nature Reserve Administration, the Dinghu Mountain National Nature Reserve Administration and the Nankun Mountain Provincial Nature Reserve Administration. The sample collection work and molecular experiments complied with local legislation, national and international guidelines, and did not involve protected species. We also abide by the Convention on the Trade in Endangered Species of Wild Fauna and Flora.

Fresh flower buds of the four species at various developmental stages were collected from plants growing in natural habitats. Anthers were carefully excised under a binocular microscope, measured, and classified according to their length and pollen development stage. The four development stages, viz. microspore mother cell (MMC) stage, tetrad (TET) stage, uninucleate microspore stage and bicellular pollen (BCP) stage, were determined by examining squash preparations stained with acetic carmine under a Zeiss AX10 light microscope (Carl Zeiss MicroImaging GmbH, Jena, Germany).

To accurately determine morphological and histological events during pollen development, the anthers of the four ginger species were examined cytologically in semi-thin sections. For the semi-thin sections, anthers were fixed in 2.5% glutaraldehyde in 0.1 M KH_2_PO_4_ buffer (pH 7.2). The samples were washed in buffer, post-fixed with 1% OsO_4_ overnight, dehydrated using an acetone series and embedded in Epon 812 resin, and then cured at 60℃. Semi-thin (1–2 μm) sections were cut with glass knives using a Leica Reichert Ultracut S ultramicrotome (Leica, Austria). To detect polysaccharides by the periodic acid-Schiff (PAS) staining method, sections were oxidized for 10 min in 0.5% periodic acid in 0.3% nitric acid, rinsed in running water for 1–2 min with a final rinse in distilled water, stained for 60 min in Schiff’s reagent, washed three times in 0.5% sodium metabisulfite for 2 min each, rinsed for 5 min in running water and then transferred to distilled water. To detect lipids, sections were rinsed for 1–2 min in 70% ethanol, stained in fresh 1% Sudan Black B in 70% ethanol for 10 min at 60℃, rinsed for 1 min in 70% ethanol, and then transferred to distilled water [94]. Sections were examined and photographed using a ZEISS AX10 research photomicroscope (Carl Zeiss MicroImaging GmbH, Jena, Germany).

### RNA extraction and sequencing

To obtain genome-wide gene expression profiles of the four ginger species with different types of pollen coat, transcriptome analysis was conducted. Total RNA of the anthers of microspore mother cell, tetrad and bicellular pollen stages was extracted using RNAprep Pure (DP441) (TIANGEN, Beijing, China). RNA integrity was assessed using the RNA Nano 6000 Assay Kit of the Bioanalyzer 2100 system (Agilent Technologies, CA, USA). The mRNA was purified from total RNA using poly-T oligo-attached magnetic beads. Double-stranded cDNA was sequenced on an Illumina Novaseq platform, and 150-bp paired-end reads were generated. These experiments were completed by Beijing Novogene Co. Ltd (https://www.novogene.com, Beijing, China).

### De novo assembly and annotation

Raw reads in fastq format were first processed through in-house Perl scripts. In this step, clean reads were obtained by removing reads containing adapter, containing poly-N, and low-quality reads from raw data. At the same time, Q20, Q30 and GC content of the clean data were calculated. All downstream analyses were based on clean data of high quality. Transcriptome assembly was accomplished using Trinity [95] with min_kmer_cov set to 2 by default and all other parameters set to default. All unigenes were annotated based on similarity to the public NCBI non-redundant protein database (NR), Swiss-Prot protein database (Swiss-Prot, http://www.expasy.ch/sprot), Kyoto Encyclopedia of Genes and Genomes (KEGG, http://www.genome.jp/kegg/) [96, 97], Cluster of Orthologous Groups database (COG, http://www.ncbi.nlm.nih.gov/COG/), Gene Ontology (GO) database and NCBI nucleotide database (NT). The expression quantity of each gene (fragments per kilobase of exon model per million mapped fragments, FPKM) was estimated by Cuffdiff software. GO and KEGG enrichment analysis of specific highly expressed genes (FPKM ≥ 3rd quartile, a criterion used to define highly expressed genes) and positively selected genes (PSGs) were implemented by the clusterProfiler R package [98] and TBtools [99].

### Identification of orthologous genes and Ka/Ks analyses

We inferred homologous protein groups among the four species of Zingiberaceae using OrthoFinder v2.3.11 [100] with a cutoff E-value set at 1e^−5^. Only one-to-one orthologs were retained for further analysis. Each orthogroup was aligned using MAFFT v7.313 [101] in codon alignment mode. The species tree was generated with IQ-tree built-in OrthoFinder using the automated parameter; 1000 bootstrap replicates were used to assess branch reliability.

The nonsynonymous (Ka) / synonymous substitution (Ks) rate (ω = Ka / Ks) is a measure of selective pressure, with values of ω > 1, = 1 and < 1 indicating positive selection, neutral selection, and purifying selection, respectively. The ω ratios were estimated using the codon-based maximum likelihood (ML) model implemented in the codeml program in PAML 4.9j [102, 103]. A well-accepted phylogeny of the four species was used as an input tree in our analysis of each gene. Branch models and branch site models were used, and ML scores were estimated for each. Positively selected genes were identified, setting Cco or Hhn as the foreground branch and the other two species (with a ‘typical’ pollen coat) as background branch in PAML. These analyses were automated using LMAP v1.0.2 [104]. We used a BLAST v2.2.28 + (e-value = 1e^−5^) search to find their orthologous genes in *Arabidopsis thaliana* for the functional analysis of these ginger orthologs. To facilitate the comparison of unigene expression among the four ginger species, all unigenes were also converted to the homologous genes in *A. thaliana*.

## Supplementary Information

**Additional file 1:** **Fig. S1.** Transverse anther sections of *Pyrgophyllum yunnanense* at different developmental stages stained with Periodic acid - Schiff (PAS, a staining method used to detect polysaccharides) and Sudan Black B (a staining method used to detect lipid), showing the distribution of polysaccharides and lipids in different anther tissues. Polysaccharides stained red, lipid stained black dots*.* MMC, microspore mother cell; P, pollen grain; EMSP, early microspores; MSP, microspores; the arrow shows the liquid in the locule. Scale bars: 50μm. **Fig. S2.** Gene ontology (GO) classification of assembled unigenes of *Caulokaempferia coenobialis* (Cco), *Hornstedtia hainanensis* (Hhn), *Pyrgophyllum yunnanense* (Pyn), and *Zingiber nudicarpum* (Znu). **Table S1.** Summary of transcriptome data for *Caulokaempferia coenobialis* (Cco), *Hornstedtia hainanensis* (Hhn), *Pyrgophyllum yunnanense* (Pyn), and *Zingiber nudicarpum* (Znu). **Table S2.** Summary of functional annotation of unigenes of *Caulokaempferia coenobialis* (Cco), *Hornstedtia hainanensis* (Hhn), *Pyrgophyllum yunnanense* (Pyn), and *Zingiber nudicarpum* (Znu). **Table S3.** KOG classification of unigenes in *Caulokaempferia coenobialis* (Cco), *Hornstedtia hainanensis* (Hhn), *Pyrgophyllum yunnanense* (Pyn), and *Zingiber nudicarpum* (Znu).were classified into 25 functional categories. **Table S4.** KEGG_classification_count of *Caulokaempferia coenobialis* (Cco), *Hornstedtia hainanensis* (Hhn), *Pyrgophyllum yunnanense* (Pyn), and *Zingiber nudicarpum* (Znu). **Table S5.** Enriched GO Biological Process (GO BP) and KEGG Pathway of specific highly expressed genes in four gingers. **Table S5-1.** Enriched GO Biological Process (GO BP) and KEGG Pathway of specific highly expressed genes in Caulokaempferia coenobialis (Cco). **Table S5-2.** Enriched GO Biological Process (GO-BP) and KEGG Pathway of specific highly expressed genes in *Hornstedtia hainanensis* (Hhn). **Table S5-3.** Enriched GO Biological Process (GO-BP) and KEGG Pathway of specific highly expressed genes in *Pyrgophyllum yunnanense* (Pyn). **Table S5-4.** Enriched GO Biological Process (GO-BP) and KEGG Pathway of specific highly expressed genes in *Zingiber nudicarpum *(Znu). **Table S6.** Detection of selection for pollen coat formation genes in *Caulokaempferia coenobialis* (Cco), *Hornstedtia hainanensis* (Hhn) Branch using Branch Model of PAML. **Table S8.** Enriched GO Biological Process (GO-BP), and KEGG Pathway of positively selected genes in *Caulokaempferia coenobialis* (Cco) and *Hornstedtia hainanensis* (Hhn) branches. **Table S8-1.** Enriched GO Biological Process (GO-BP), and KEGG Pathway of positively selected genes in *Caulokaempferia coenobialis* (Cco) branch. **Table S8-2.** Enriched GO Biological Process (GO-BP), and KEGG Pathway of positively selected genes in *Hornstedtia hainanensis* (Hhn) branch. **Table S9.** Candidates genes of *Caulokaempferia coenobialis* and *Hornstedtia hainanensis* involved in mucilage-like or gum-like pollen coat formation. **Table S9-1.** Candidates' genes of *Caulokaempferia coenobialis* involved in mucilage-like pollen coat formation. **Table S9-2.** Candidates' genes of *Hornstedtia hainanensis* involved in mucilage-like pollen coat formation. **Table S10.** Expression pattern of some genes related to pollen wall formation in four gingers, *Caulokaempferia coenobialis* (Cco), *Hornstedtia hainanensis *(Hhn), *Pyrgophyllum yunnanense *(Pyn), *Zingiber nudicarpum *(Znu) and their homologs in *Arabidopsis thaliana *(At).

**Additional file 2:** **Table S7.** Detection of selection for pollen coat formation genes in Caulokaempferia coenobialis (Cco), Hornstedtia hainanensis (Hhn) Branch using Branch-site Model of PAML.

## Data Availability

The sequencing reads were submitted to the NCBI SRA and can be accessed via NCBI BioProject accession number PRJNA793275 (https://www.ncbi.nlm.nih.gov/bioproject/PRJNA793275/).

## Abbreviations

BCP: Bicellular pollen
BP: Biological process
Cco: *Caulokaempferia coenobialis*
FPKM: Fragments per kilobase of exon model per million mapped fragments
GO: Gene ontology
Hhn: *Hornstedtia hainanensis*
Ka: Nonsynonymous substitution
KEGG: Kyoto encyclopedia of genes and genomes
Ks: Synonymous substitution
MMC: Microspore mother cell
MSP: Microspores
P: Pollen grain
Pyn: *Pyrgophyllum yunnanense*
SHE: Specific highly expressed genes
T: Tapetum
TET: Tetrad
Znu: *Zingiber nudicarpum*
