# Supplementary material for: Cytochemical and comparative transcriptome analyses elucidate the formation and ecological adaptation of three types of pollen coat in Zingiberaceae
Source: BMC Plant Biol. 2022 Aug 20;22:407. doi: 10.1186/s12870-022-03796-2 (PMC9392269; doi:10.1186/s12870-022-03796-2)
Supplement: Supplementary file 1 — Additional file 1: Fig. S1. Transverse anther sections of Pyrgophyllum yunnanense at different developmental stages stained with Periodic acid - Schiff (PAS, a staining method used to detect polysaccharides) and Sudan Black B (a staining method used to detect lipid), showing the distribution of polysaccharides and lipids in different anther tissues. Polysaccharides stained red, lipid stained black dots. MMC, microspore mother cell; P, pollen grain; EMSP, early microspores; MSP, microspores; the arrow shows the liquid in the locule. Scale bars: 50μm. Fig. S2. Gene ontology (GO) classification of assembled unigenes of Caulokaempferia coenobialis (Cco), Hornstedtia hainanensis (Hhn), Pyrgophyllum yunnanense (Pyn), and Zingiber nudicarpum (Znu). Table S1. Summary of transcriptome data for Caulokaempferia coenobialis (Cco), Hornstedtia hainanensis (Hhn), Pyrgophyllum yunnanense (Pyn), and Zingiber nudicarpum (Znu). Table S2. Summary of functional annotation of unigenes of Caulokaempferia coenobialis (Cco), Hornstedtia hainanensis (Hhn), Pyrgophyllum yunnanense (Pyn), and Zingiber nudicarpum (Znu). Table S3. KOG classification of unigenes in Caulokaempferia coenobialis (Cco), Hornstedtia hainanensis (Hhn), Pyrgophyllum yunnanense (Pyn), and Zingiber nudicarpum (Znu).were classified into 25 functional categories. Table S4. KEGG_classification_count of Caulokaempferia coenobialis (Cco), Hornstedtia hainanensis (Hhn), Pyrgophyllum yunnanense (Pyn), and Zingiber nudicarpum (Znu). Table S5. Enriched GO Biological Process (GO BP) and KEGG Pathway of specific highly expressed genes in four gingers. Table S5-1. Enriched GO Biological Process (GO BP) and KEGG Pathway of specific highly expressed genes in Caulokaempferia coenobialis (Cco). Table S5-2. Enriched GO Biological Process (GO-BP) and KEGG Pathway of specific highly expressed genes in Hornstedtia hainanensis (Hhn). Table S5-3. Enriched GO Biological Process (GO-BP) and KEGG Pathway of specific highly expressed genes in Pyr [file 12870_2022_3796_MOESM1_ESM.docx]

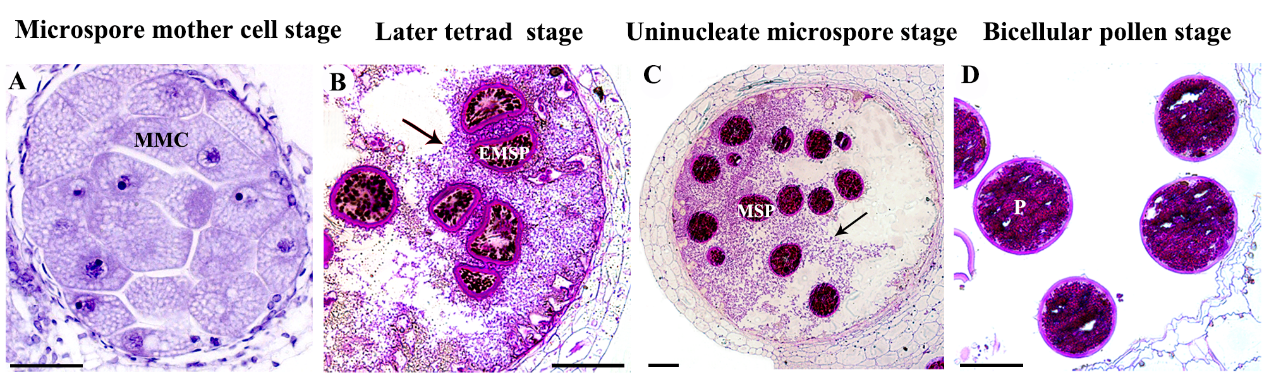


Fig. S1 Transverse anther sections of *Pyrgophyllum yunnanense* at different developmental stages stained with Periodic acid - Schiff (PAS, a staining method used to detect polysaccharides) and Sudan Black B (a staining method used to detect lipid), showing the distribution of polysaccharides and lipids in different anther tissues. Polysaccharides stained red, lipid stained black dots*.* MMC, microspore mother cell; P, pollen grain; EMSP, early microspores; MSP, microspores; the arrow shows the liquid in the locule. Scale bars: 50μm.


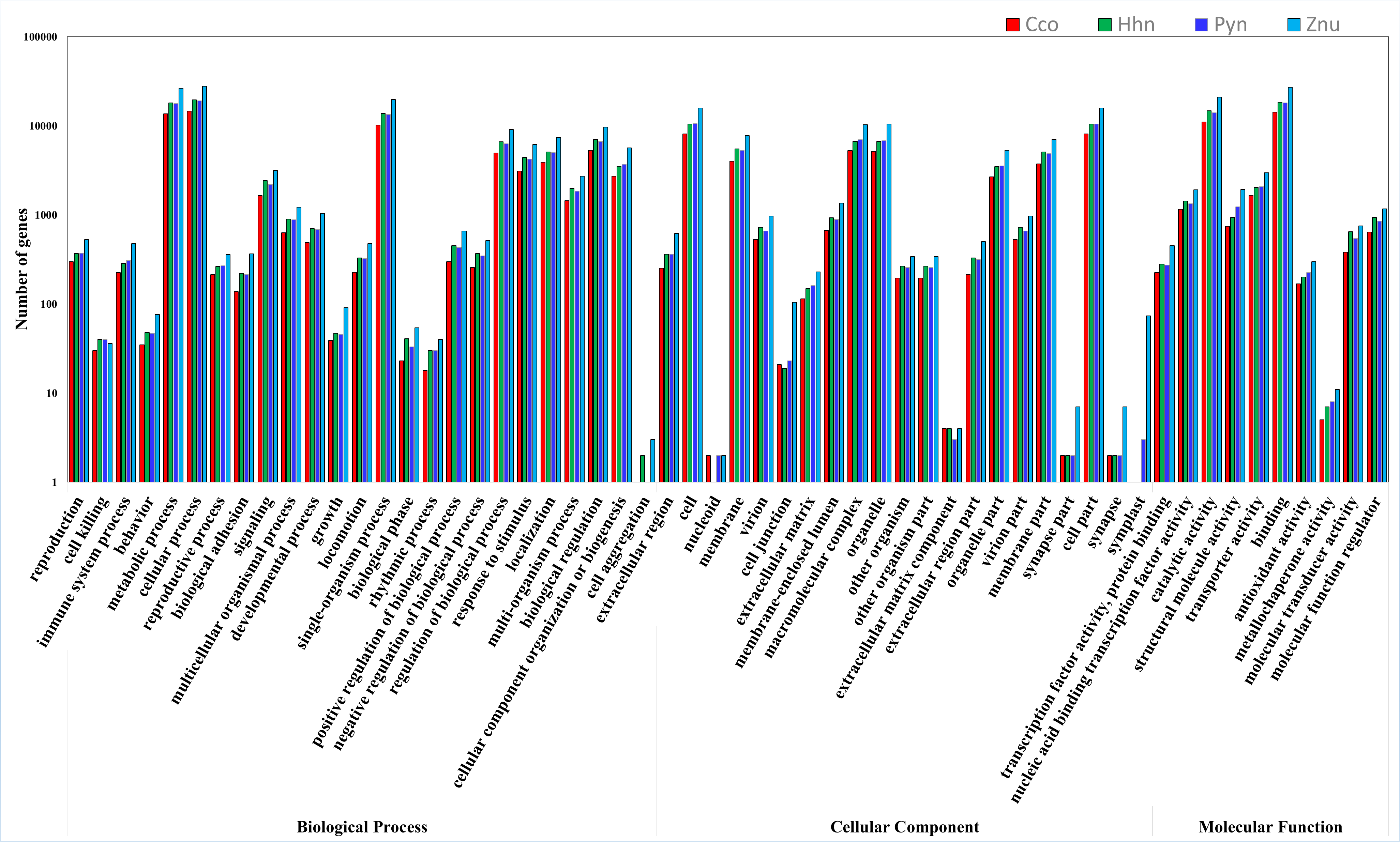


Fig. S2. Gene ontology (GO) classification of assembled unigenes of *Caulokaempferia coenobialis* (Cco), *Hornstedtia hainanensis* (Hhn), *Pyrgophyllum yunnanense* (Pyn), and *Zingiber nudicarpum* (Znu). Results are summarized in three main categories: biological process, cellular component, and molecular function. The x-axis indicates the subgroups in GO annotation while the y-axis indicates the number of specific categories of genes in each main category.

Table S1 Summary of transcriptome data for *Caulokaempferia coenobialis* (Cco), *Hornstedtia hainanensis* (Hhn), *Pyrgophyllum yunnanense* (Pyn), and *Zingiber nudicarpum* (Znu)

|  | Cco | Hhn | Pyn | Znu |
| --- | --- | --- | --- | --- |
| Total number of unigenes | 103500 | 155568 | 140315 | 174594 |
| Mean length of unigenes | 721 | 763 | 698 | 682 |
| Median length of unigenes | 396 | 428 | 389 | 357 |
| N50 value of unigenes | 1186 | 1213 | 1098 | 1143 |
| Length range of unigenes | 201-16885 | 201-16891 | 201-15782 | 201-14827 |
| Unigenes with annotation | 40660 (39.28%) | 59990 (38.56%) | 56882 (40.53%) | 96876 (55.48%) |

Table S2 Summary of functional annotation of unigenes of *Caulokaempferia coenobialis* (Cco), *Hornstedtia hainanensis* (Hhn), *Pyrgophyllum yunnanense* (Pyn), and *Zingiber nudicarpum* (Znu).

| Annotation database | Unigene number and percentage | | | |
| --- | --- | --- | --- | --- |
|  | Cco | Hhn | Pyn | Znu |
| Annotated in NR | 35065 (33.87) | 41277 (26.53) | 42634 (30.38) | 68740 (39.37) |
| Annotated in NT | 21445 (20.71) | 34307 (22.05) | 31716 (22.60) | 70164 (40.18) |
| Annotated in KO | 12860 (12.42) | 14444 (9.28) | 16511 (11.76) | 27381 (15.68) |
| Annotated in SwissProt | 25046 (24.19) | 35324 (22.70) | 34064 (24.27) | 56719 (32.48) |
| Annotated in PFAM | 25792 (24.91) | 34905 (22.43) | 33858 (24.12) | 50052 (28.66) |
| Annotated in GO | 25883 (25.00) | 35108 (22.56) | 34186 (24.36) | 50670 (29.02) |
| Annotated in KOG | 7634 (7.37) | 10081 (6.48) | 11431 (8.14) | 18957 (10.85) |
| Annotated in all Databases | 4102 (3.96) | 4943 (3.17) | 5658 (4.03) | 9979 (5.71) |
| Annotated in at least one Database | 40660 (39.28) | 59990 (38.56) | 56882 (40.53) | 96876 (55.48) |
| Total Unigenes | 103500 (100) | 155568 (100) | 140315 (100) | 174594 (100) |

Table S3 KOG classification of unigenes in *Caulokaempferia coenobialis* (Cco), *Hornstedtia hainanensis* (Hhn), *Pyrgophyllum yunnanense* (Pyn), and *Zingiber nudicarpum* (Znu).were classified into 25 functional categories

| Class | Abbreviation | Cco | Hhn | Pyn | Znu |
| --- | --- | --- | --- | --- | --- |
| Posttranslational modification, protein turnover, chaperones | O | 1154(13.55%) | 1405(12.48%) | 1741(13.80%) | 2994(14.23%) |
| General function prediction only | R | 925(10.86%) | 1161(10.32%) | 1175(9.31%) | 2080(9.89%) |
| Translation, ribosomal structure and biogenesis | J | 789(9.26%) | 947(8.41%) | 1513(11.99%) | 2494(11.86%) |
| Intracellular trafficking, secretion, and vesicular transport | U | 559(6.56%) | 674(5.99%) | 787(6.24%) | 1402(6.66%) |
| Signal transduction mechanisms | T | 545(6.40%) | 1068(9.49%) | 865(6.86%) | 1471(6.99%) |
| RNA processing and modification | A | 513(6.02%) | 619(5.50%) | 732(5.80%) | 1159(5.51%) |
| Function unknown | S | 479(5.62%) | 499(4.43%) | 569(4.51%) | 824(3.92%) |
| Energy production and conversion | C | 433(5.08%) | 565(5.02%) | 800(6.34%) | 1294(6.15%) |
| Transcription | K | 395(4.64%) | 583(5.18%) | 540(4.28%) | 931(4.43%) |
| Carbohydrate transport and metabolism | G | 372(4.37%) | 485(4.31%) | 554(4.39%) | 906(4.31%) |
| Amino acid transport and metabolism | E | 340(3.99%) | 424(3.77%) | 427(3.38%) | 800(3.80%) |
| Lipid transport and metabolism | I | 337(3.96%) | 365(3.24%) | 437(3.46%) | 752(3.57%) |
| Replication, recombination and repair | L | 240(2.82%) | 415(3.69%) | 339(2.69%) | 505(2.40%) |
| Cell cycle control, cell division, chromosome partitioning | D | 235(2.76%) | 330(2.93%) | 322(2.55%) | 485(2.31%) |
| Cytoskeleton | Z | 222(2.61%) | 369(3.28%) | 506(4.01%) | 722(3.43%) |
| Inorganic ion transport and metabolism | P | 200(2.35%) | 242(2.15%) | 272(2.16%) | 447(2.12%) |
| Nucleotide transport and metabolism | F | 178(2.09%) | 291(2.59%) | 238(1.89%) | 417(1.98%) |
| Chromatin structure and dynamics | B | 147(1.73%) | 210(1.87%) | 210(1.66%) | 331(1.57%) |
| Coenzyme transport and metabolism | H | 141(1.66%) | 173(1.54%) | 172(1.36%) | 302(1.44%) |
| Secondary metabolites biosynthesis, transport and catabolism | Q | 122(1.43%) | 176(1.56%) | 136(1.08%) | 250(1.19%) |
| Cell wall/membrane/envelope biogenesis | M | 93(1.09%) | 99(0.88%) | 113(0.90%) | 165(0.78%) |
| Defense mechanisms | V | 44(0.52%) | 53(0.47%) | 60(0.48%) | 93(0.44%) |
| Nuclear structure | Y | 41(0.48%) | 61(0.54%) | 56(0.44%) | 102(0.48%) |
| Extracellular structures | W | 8(0.09%) | 34(0.30%) | 46(0.36%) | 98(0.47%) |
| Cell motility | N | 7(0.08%) | 7(0.06%) | 6(0.05%) | 12(0.06%) |

Table S4 KEGG_classification_count of *Caulokaempferia coenobialis* (Cco), *Hornstedtia hainanensis* (Hhn), *Pyrgophyllum yunnanense* (Pyn), and *Zingiber nudicarpum* (Znu).

| Pathway Hierarchy1 | Pathway Hierarchy2 | Cco | Hhn | Pyn | Znu |
| --- | --- | --- | --- | --- | --- |
| Metabolism | Carbohydrate metabolism | 1217 | 1377 | 1575 | 2668 |
| Genetic Information Processing | Translation | 1032 | 1161 | 1770 | 3049 |
| Genetic Information Processing | Folding, sorting and degradation | 906 | 1019 | 1270 | 2185 |
| Cellular Processes | Transport and catabolism | 708 | 781 | 923 | 1592 |
| Metabolism | Overview | 705 | 871 | 1010 | 1937 |
| Organismal Systems | Environmental adaptation | 688 | 801 | 812 | 1234 |
| Metabolism | Energy metabolism | 605 | 659 | 983 | 1746 |
| Metabolism | Lipid metabolism | 593 | 670 | 716 | 1241 |
| Metabolism | Amino acid metabolism | 590 | 711 | 829 | 1530 |
| Environmental Information Processing | Signal transduction | 556 | 592 | 598 | 950 |
| Genetic Information Processing | Transcription | 379 | 479 | 549 | 1010 |
| Metabolism | Biosynthesis of other secondary metabolites | 360 | 427 | 469 | 761 |
| Metabolism | Metabolism of cofactors and vitamins | 317 | 367 | 375 | 636 |
| Metabolism | Nucleotide metabolism | 306 | 474 | 427 | 717 |
| Metabolism | Metabolism of terpenoids and polyketides | 305 | 353 | 397 | 573 |
| Metabolism | Metabolism of other amino acids | 243 | 304 | 345 | 682 |
| Genetic Information Processing | Replication and repair | 175 | 249 | 251 | 384 |
| Metabolism | Glycan biosynthesis and metabolism | 175 | 174 | 209 | 330 |
| Environmental Information Processing | Membrane transport | 56 | 103 | 53 | 122 |

Table S5. Enriched GO Biological Process (GO BP) and KEGG Pathway of specific highly expressed genes in four gingers

Table S5-1 Enriched GO Biological Process (GO BP) and KEGG Pathway of specific highly expressed genes in Caulokaempferia coenobialis (Cco)

| Term | *P-*value |
| --- | --- |
| GO-BP: Pectin catabolic process | 3.51E-03 |
| GO-BP: Cell wall modification | 8.01E-03 |
| GO-BP: Polysaccharide catabolic process | 1.23E-02 |
| GO-BP: Polysaccharide metabolic process | 1.32E-02 |
| GO-BP: Response to nematode | 2.77E-02 |
| GO-BP: Pectin metabolic process | 2.77E-02 |
| GO-BP: Galacturonan metabolic process | 2.77E-02 |
| GO-BP: Supramolecular fiber organization | 3.07E-02 |
| KEGG: Galactose metabolism | 7.31E-03 |
| KEGG: MAPK signaling pathway plant | 9.59E-03 |
| KEGG: Carbohydrate metabolism | 1.51E-02 |
| KEGG: Pentose and glucuronate interconversions | 1.58E-02 |
| KEGG: Environmental Information Processing | 2.39E-02 |
| KEGG: Starch and sucrose metabolism | 3.52E-02 |
| KEGG: Protein families signaling and cellular processes | 4.37E-02 |
| KEGG: Homologous recombination | 4.42E-02 |
| KEGG: Ubiquitin mediated proteolysis | 4.61E-02 |
| KEGG: Plant pathogen interaction | 4.61E-02 |
| KEGG: Endocytosis | 4.76E-02 |

GO categories using q value less than 0.05 as cutoffs

Table S5-2 Enriched GO Biological Process (GO-BP) and KEGG Pathway of specific highly expressed genes in *Hornstedtia hainanensis* (Hhn)

| Term | *P-*value |
| --- | --- |
| GO-BP: Photosynthesis | 2.40E-04 |
| GO-BP: Double-strand break repair via homologous recombination | 2.89E-03 |
| GO-BP: Recombinational repair | 3.34E-03 |
| GO-BP: DNA repair | 3.34E-03 |
| GO-BP: DNA recombination | 4.34E-03 |
| GO-BP: Cell cycle process | 4.34E-03 |
| GO-BP: Organelle fission | 4.34E-03 |
| GO-BP: Meiotic cell cycle | 5.63E-03 |
| GO-BP: Nuclear division | 7.05E-03 |
| GO-BP: Cellular response to DNA damage stimulus | 7.05E-03 |
| GO-BP: Double-strand break repair | 7.05E-03 |
| GO-BP: Photosynthesis, light reaction | 1.19E-02 |
| GO-BP: Generation of precursor metabolites and energy | 1.32E-02 |
| GO-BP: Divalent metal ion transport | 2.00E-02 |
| GO-BP: Mismatch repair | 2.05E-02 |
| GO-BP: Divalent inorganic cation transport | 2.05E-02 |
| GO-BP: Ribosome biogenesis | 2.05E-02 |
| GO-BP: Virus induced gene silencing | 2.05E-02 |
| GO-BP: Modulation by symbiont of RNA levels in host | 2.05E-02 |
| GO-BP: Modulation of RNA levels in other organism involved in symbiotic interaction | 2.05E-02 |
| GO-BP: Floral whorl development | 2.05E-02 |
| GO-BP: Response to cold | 2.32E-02 |
| GO-BP: Interaction with host | 2.48E-02 |
| GO-BP: Cellular response to virus | 2.48E-02 |
| GO-BP: Chloroplast RNA processing | 2.57E-02 |
| GO-BP: Multicellular organismal reproductive process | 2.72E-02 |
| GO-BP: Anther development | 2.72E-02 |
| GO-BP: Flower development | 2.72E-02 |
| GO-BP: RNA modification | 3.20E-02 |
| GO-BP: Multicellular organism reproduction | 3.48E-02 |
| GO-BP: Negative regulation of cell cycle | 3.60E-02 |
| GO-BP: Galactose metabolic process | 3.60E-02 |
| GO-BP: Monocarboxylic acid transport | 3.60E-02 |
| GO-BP: Calcium ion transport | 3.60E-02 |
| GO-BP: Meiotic nuclear division | 3.69E-02 |
| GO-BP: Chlorophyll metabolic process | 3.69E-02 |
| GO-BP: Reductive pentose-phosphate cycle | 3.69E-02 |

Table S5-2 Enriched GO Biological Process (GO-BP) and KEGG Pathway of specific highly expressed genes in *Hornstedtia hainanensis* (Hhn) (Continued)

| Term | *P-*value |
| --- | --- |
| GO-BP: Histone H3-K27 methylation | 3.69E-02 |
| GO-BP: ncRNA metabolic process | 3.69E-02 |
| GO-BP: Hexose metabolic process | 4.01E-02 |
| GO-BP: Cellular amino acid metabolic process | 4.24E-02 |
| GO-BP: Photosynthesis, dark reaction | 4.26E-02 |
| GO-BP: Meiotic cell cycle process | 4.26E-02 |
| GO-BP: Floral organ development | 4.31E-02 |
| GO-BP: Stem cell population maintenance | 4.70E-02 |
| GO-BP: Maintenance of cell number | 4.70E-02 |
| GO-BP: Carbon fixation | 4.70E-02 |
| GO-BP: Modulation by symbiont of host process | 4.70E-02 |
| GO-BP: Response to ionizing radiation | 4.70E-02 |
| KEGG: DNA repair and recombination proteins | 7.29E-05 |
| KEGG: Homologous recombination | 4.26E-03 |
| KEGG: Galactose metabolism | 8.10E-03 |
| KEGG: Replication and repair | 9.14E-03 |
| KEGG: Mismatch repair | 1.64E-02 |
| KEGG: Porphyrin and chlorophyll metabolism | 2.02E-02 |
| KEGG: Protein families: signaling and cellular processes | 2.46E-02 |
| KEGG: Nitrogen metabolism | 2.97E-02 |
| KEGG: Ion channels | 3.23E-02 |
| KEGG: Organismal Systems | 4.01E-02 |
| KEGG: Environmental adaptation | 4.01E-02 |
| KEGG: Fatty acid degradation | 4.15E-02 |
| KEGG: Metabolism of terpenoids and polyketides | 4.86E-02 |

GO categories using q value less than 0.05 as cutoffs

Table S5-3 Enriched GO Biological Process (GO-BP) and KEGG Pathway of specific highly expressed genes in *Pyrgophyllum yunnanense* (Pyn)

| Term | *P*-value |
| --- | --- |
| no GO-BP terms enriched |  |
| KEGG: Protein families: signaling and cellular processes | 6.56E-03 |
| KEGG: Transporters | 9.79E-03 |
| KEGG: Amino acid metabolism | 1.16E-02 |
| KEGG: Biosynthesis of other secondary metabolites | 1.82E-02 |
| KEGG: Starch and sucrose metabolism | 2.85E-02 |
| KEGG: Metabolism | 3.40E-02 |

GO categories using q value less than 0.05 as cutoffs

Table S5-4 Enriched GO Biological Process (GO-BP) and KEGG Pathway of specific highly expressed genes in *Zingiber nudicarpum* (Znu)

| Term | *P-*value |
| --- | --- |
| GO-BP: Secondary metabolic process | 1.47E-02 |
| GO-BP: DNA conformation change | 1.47E-02 |
| GO-BP: Response to cadmium ion | 1.47E-02 |
| GO-BP: Chiasma assembly | 1.90E-02 |
| GO-BP: Double-strand break repair | 1.90E-02 |
| GO-BP: Response to metal ion | 2.49E-02 |
| GO-BP: Double-strand break repair via homologous recombination | 2.49E-02 |
| GO-BP: DNA packaging | 2.49E-02 |
| GO-BP: Recombinational repair | 3.36E-02 |
| GO-BP: Defense response to Gram-negative bacterium | 3.36E-02 |
| GO-BP: DNA recombination | 3.36E-02 |
| GO-BP: Nucleosome assembly | 3.89E-02 |
| GO-BP: Mitotic cell cycle | 3.89E-02 |
| GO-BP: Leaf development | 3.89E-02 |
| GO-BP: Protein-DNA complex assembly | 3.89E-02 |
| GO-BP: Cell division | 3.89E-02 |
| GO-BP: Nucleosome organization | 4.24E-02 |
| GO-BP: Protein-DNA complex subunit organization | 4.24E-02 |
| GO-BP: Chromatin assembly | 4.92E-02 |
| KEGG: Biosynthesis of other secondary metabolites | 4.20E-05 |
| KEGG: Chromosome and associated proteins | 7.11E-05 |
| KEGG: Zeatin biosynthesis | 3.76E-04 |
| KEGG: Phenylpropanoid biosynthesis | 4.35E-03 |
| KEGG: DNA replication proteins | 8.73E-03 |
| KEGG: Ribosome | 9.22E-03 |
| KEGG: Protein families: signaling and cellular processes | 1.67E-02 |
| KEGG: Tropane, piperidine and pyridine alkaloid biosynthesis | 1.92E-02 |
| KEGG: Glyoxylate and dicarboxylate metabolism | 2.16E-02 |
| KEGG: Metabolism | 2.49E-02 |
| KEGG: Tyrosine metabolism | 3.20E-02 |
| KEGG: Starch and sucrose metabolism | 3.66E-02 |
| KEGG: alpha-Linolenic acid metabolism | 3.83E-02 |
| KEGG: Transporters | 4.60E-02 |
| KEGG: Carbohydrate metabolism | 4.62E-02 |

GO categories using q value less than 0.05 as cutoffs

Table S6 Detection of selection for pollen coat formation genes in *Caulokaempferia coenobialis* (Cco), *Hornstedtia hainanensis* (Hhn) Branch using Branch Model of PAML

| Branch | Gene | orthologs in Arabidopsis | -ln L | P-value (fdr) | Annotation | Putative function(s) |
| --- | --- | --- | --- | --- | --- | --- |
| Cco | c62618_g2 | AT5G28290 | -413.450234 | 1.10E-02 | Encodes AtNek3, a member of the NIMA-related serine/threonine kinases (Neks) that have been linked to cell-cycle regulation in fungi and mammals. Plant Neks might be involved in plant development processes. |  |
|  | c11698_g1 | AT4G30360 | -971.493967 | 4.61E-21 | member of Cyclic nucleotide gated channel family |  |
|  | c51826_g2 | AT4G15080 | -3404.77833 | 1.17E-51 | DHHC-type zinc finger family protein;(source:Araport11) | transport |
|  | c90986_g1 | AT4G00360 | -224.710109 | 6.10E-04 | Encodes a member of the CYP86A subfamily of cytochrome p450 genes. Expressed at moderate levels in flowers, leaves, roots and stems. |  |
|  | c74617_g1 | AT3G53510 | -311.045904 | 2.06E-02 | Belongs to a clade of five Arabidopsis thaliana ABCG half-transporters that are required for synthesis of an effective suberin barrier in roots and seed coats (ABCG2, ABCG6, and ABCG20) and for synthesis of an intact pollen wall (ABCG1 and ABCG16). Phloem-expressed and plasma membrane-localized jasmonate transporter which together with JAT3 and GLR3.3 involved in regulating long-distance translocation of JA, which is important for driving the loading, translocation of JA in the phloem pathway by a self-propagation mode, contributing to wound-induced systemic response/resistance. | transport ABCG20 |

Table S6 Detection of selection for pollen coat formation genes in *Caulokaempferia coenobialis* (Cco), *Hornstedtia hainanensis* (Hhn) Branch using Branch Model of PAML (continued)

| Branch | Gene | orthologs in Arabidopsis | -ln L | P-value (fdr) | Annotation | Putative function(s) |
| --- | --- | --- | --- | --- | --- | --- |
| Cco | c31821_g1 | AT3G14680 | -860.193312 | 4.25E-02 | putative cytochrome P450 |  |
|  | c73851_g1 | AT3G02850 | -2372.0132 | 6.27E-14 | Encodes SKOR, a member of Shaker family potassium ion (K+) channel. This family includes five groups based on phylogenetic analysis (FEBS Letters (2007) 581: 2357): I (inward rectifying channel): AKT1 (AT2G26650), AKT5 (AT4G32500) and SPIK (also known as AKT6, AT2G25600); II (inward rectifying channel): KAT1 (AT5G46240) and KAT2 (AT4G18290); III (weakly inward rectifying channel): AKT2 (AT4G22200); IV (regulatory subunit involved in inwardly rectifying conductance formation): KAT3 (also known as AtKC1, AT4G32650); V (outward rectifying channel): SKOR (AT3G02850) and GORK (AT5G37500). Mediates the delivery of K+ from stelar cells to the xylem in the roots towards the shoot. mRNA accumulation is modulated by abscisic acid. K+ gating activity is modulated by external and internal K+. Involved in response to low potassium. | transport |
|  | c55570_g1 | AT2G41900 | -3473.28841 | 3.57E-40 | AtOXS2 specifcally entered the nuclear under salt stress. The specific nuclear localization of AtOXS2 could play a role in salt tolerance at the molecular level. These results implied that AtOXS2 might target some downstream cis-elements which are required for salt stress responses | Transcription |
|  | c16484_g2 | AT1G23090 | -893.333497 | 4.35E-03 | Encodes AST91 mRNA for sulfate transporter. | transport |

Table S6 Detection of selection for pollen coat formation genes in *Caulokaempferia coenobialis* (Cco), *Hornstedtia hainanensis* (Hhn) Branch using Branch Model of PAML (continued)

| Branch | Gene | orthologs in Arabidopsis | -ln *L* | *P*-value (fdr) | Annotation | Putative function(s) |
| --- | --- | --- | --- | --- | --- | --- |
| Cco | c62207_g2 | AT1G04990 | -2092.50781 | 1.67E-03 | Zinc finger C-x8-C-x5-C-x3-H type family protein;(source:Araport11) | Transcription |
|  | c3271_g1 |  | -1592.02368 | 8.76E-03 |  |  |
|  | c37838_g1 |  | -326.935798 | 2.37E-02 |  |  |
|  | c62792_g1 |  | -16356.6637 | 2.91E-241 |  |  |
|  | c44433_g1 |  | -1167.29937 | 8.28E-17 |  |  |
|  | c93250_g1 |  | -205.126696 | 3.47E-03 |  |  |
|  | c99482_g1 |  | -604.379176 | 5.29E-05 |  |  |
|  | c83166_g1 |  | -378.168956 | 1.05E-06 |  |  |
|  | c4257_g1 |  | -711.321611 | 1.87E-02 |  |  |
|  |  |  |  |  |  |  |
| Hhn | c81006_g1 | AT5G28290 | -413.450236 | 2.38E-05 | Encodes AtNek3, a member of the NIMA-related serine/threonine kinases (Neks) that have been linked to cell-cycle regulation in fungi and mammals. Plant Neks might be involved in plant development processes. |  |
|  | c15019_g1 | AT5G06090 | -519.463074 | 4.78E-03 | putative sn-glycerol-3-phosphate 2-O-acyltransferase，involved in CDP-diacylglycerol biosynthetic process, cutin and suberin biosynthetic process |  |
|  | c92468_g1 | AT4G35350 | -107.689811 | 3.07E-02 | tracheary element vacuolar protein |  |
|  | c63118_g1 | AT4G13650 | -1572.35089 | 1.52E-06 | Pentatricopeptide repeat (PPR) superfamily protein;(source: Araport11) |  |
|  | c19830_g2 | AT4G02700 | -893.333497 | 2.47E-02 | sulfate transporter 3;(source:Araport11) | transport |

Table S6 Detection of selection for pollen coat formation genes in *Caulokaempferia coenobialis* (Cco), *Hornstedtia hainanensis* (Hhn) Branch using Branch Model of PAML (continued)

| Branch | Gene | orthologs in Arabidopsis | -ln L | P-value (fdr) | Annotation | Putative function(s) |
| --- | --- | --- | --- | --- | --- | --- |
| Hhn | c120589_g1 | AT4G00360 | -224.710106 | 5.52E-04 | Encodes a member of the CYP86A subfamily of cytochrome p450 genes. Expressed at moderate levels in flowers, leaves, roots and stems. It is related to suberin and cutin biosynthesis |  |
|  | c65063_g1 | AT3G46870 | -614.060408 | 3.13E-02 | Pentatricopeptide repeat (PPR) superfamily protein;(source: Araport11) |  |
|  | c54822_g1 | AT3G16500 | -2048.2249 | 3.59E-23 | phytochrome-associated protein 1 (PAP1) |  |
|  | c45170_g2 | AT3G02850 | -2365.54968 | 1.39E-15 | Encodes SKOR, a member of Shaker family potassium ion (K+) channel. This family includes five groups based on phylogenetic analysis (FEBS Letters (2007) 581: 2357): I (inward rectifying channel): AKT1 (AT2G26650), AKT5 (AT4G32500) and SPIK (also known as AKT6, AT2G25600); II (inward rectifying channel): KAT1 (AT5G46240) and KAT2 (AT4G18290); III (weakly inward rectifying channel): AKT2 (AT4G22200); IV (regulatory subunit involved in inwardly rectifying conductance formation): KAT3 (also known as AtKC1, AT4G32650); V (outward rectifying channel): SKOR (AT3G02850) and GORK (AT5G37500). Mediates the delivery of K+ from stelar cells to the xylem in the roots towards the shoot. mRNA accumulation is modulated by abscisic acid. K+ gating activity is modulated by external and internal K+. Involved in response to low potassium. | transport |

Table S6 Detection of selection for pollen coat formation genes in *Caulokaempferia coenobialis* (Cco), *Hornstedtia hainanensis* (Hhn) Branch using Branch Model of PAML (continued)

| Branch | Gene | orthologs in Arabidopsis | -ln *L* | *P*-value (fdr) | Annotation | Putative function(s) |
| --- | --- | --- | --- | --- | --- | --- |
| Hhn | c35447_g1 | AT2G41900 | -3532.16282 | 1.17E-14 | AtOXS2 specifcally entered the nuclear under salt stress. The specific nuclear localization of AtOXS2 could play a role in salt tolerance at the molecular level. These results implied that AtOXS2 might target some downstream cis-elements which are required for salt stress responses |  |
|  | c55055_g1 | AT1G64780 | -1933.78877 | 3.22E-04 | encodes an ammonium transporter protein believed to act as a high affinity transporter. It is expressed in the root, primarily in endodermal and cortical cells, and contributes to ammonium uptake in the root. | transport |
|  | c39068_g1 | AT1G12500 | -1413.42124 | 8.34E-03 | Nucleotide-sugar transporter family protein;(source:Araport11) | transport |
|  | c18978_g2 |  | -386.63468 | 2.39E-02 |  |  |
|  | c61860_g2 |  | -985.001263 | 8.75E-03 |  |  |
|  | c12597_g1 |  | -661.428813 | 4.60E-03 |  |  |
|  | c41337_g5 |  | -443.331383 | 4.46E-02 |  |  |
|  | c27535_g1 |  | -681.19676 | 4.27E-02 |  |  |
|  | c107215_g1 |  | -681.003451 | 8.18E-14 |  |  |
|  | c40371_g1 |  | -327.705873 | 3.71E-03 |  |  |
|  | c133338_g1 |  | -880.626897 | 1.32E-25 |  |  |
|  | c124796_g1 |  | -205.126696 | 1.91E-02 |  |  |
|  | c67070_g1 |  | -16275.6323 | 1.31E-206 |  |  |
|  | c55111_g2 |  | -944.839529 | 1.32E-19 |  |  |

Table S8 Enriched GO Biological Process (GO-BP), and KEGG Pathway of positively selected genes in *Caulokaempferia coenobialis* (Cco) and *Hornstedtia hainanensis* (Hhn) branches

Table S8-1 Enriched GO Biological Process (GO-BP), and KEGG Pathway of positively selected genes in *Caulokaempferia coenobialis* (Cco) branch

| Term | *P*-value |
| --- | --- |
| GO-BP: Regulation of biological quality | 2.61E-04 |
| GO-BP: Vesicle-mediated transport | 8.45E-04 |
| GO-BP: Cellular localization | 1.18E-03 |
| GO-BP: Macromolecule localization | 1.40E-03 |
| GO-BP: Nodulation | 1.48E-03 |
| GO-BP: Retrograde transport, endosome to Golgi | 2.93E-03 |
| GO-BP: C21-steroid hormone metabolic process | 4.08E-03 |
| GO-BP: Androgen metabolic process | 4.08E-03 |
| GO-BP: Estrogen metabolic process | 4.08E-03 |
| GO-BP: Methylation | 4.83E-03 |
| GO-BP: Protein alkylation | 5.41E-03 |
| GO-BP: Protein methylation | 5.41E-03 |
| GO-BP: Cytosolic transport | 7.44E-03 |
| GO-BP: Protein localization | 7.64E-03 |
| GO-BP: Cellular hormone metabolic process | 7.95E-03 |
| GO-BP: Macromolecule methylation | 8.42E-03 |
| GO-BP: C-terminal protein methylation | 8.85E-03 |
| GO-BP: Post-translational protein modification | 8.85E-03 |
| GO-BP: Endosomal transport | 8.85E-03 |
| GO-BP: C-terminal protein amino acid modification | 8.85E-03 |
| GO-BP: Endoplasmic reticulum organization | 9.26E-03 |
| GO-BP: Cellular macromolecule localization | 1.04E-02 |
| GO-BP: Cellular protein localization | 1.04E-02 |
| GO-BP: Regulation of hormone levels | 1.04E-02 |
| GO-BP: Hormone metabolic process | 1.04E-02 |
| GO-BP: Response to acidic pH | 1.21E-02 |
| GO-BP: Response to pH | 1.21E-02 |
| GO-BP: Intracellular transport | 1.39E-02 |
| GO-BP: Organic substance transport | 1.41E-02 |
| GO-BP: Establishment of localization in cell | 1.43E-02 |
| GO-BP: Cellular homeostasis | 1.51E-02 |
| GO-BP: Secretion by cell | 1.53E-02 |
| GO-BP: Regulation of translation | 1.55E-02 |
| GO-BP: Regulation of cellular amide metabolic process | 1.55E-02 |
| GO-BP: Establishment of protein localization | 1.66E-02 |

Table S8-1 Enriched GO Biological Process (GO-BP), and KEGG Pathway of positively selected genes in *Caulokaempferia coenobialis* (Cco) branch (continued)

| Term | *P*-value |
| --- | --- |
| GO-BP: Exocytosis | 1.67E-02 |
| GO-BP: Secretion | 1.69E-02 |
| GO-BP: Posttranscriptional regulation of gene expression | 1.72E-02 |
| GO-BP: GPI anchor metabolic process | 1.74E-02 |
| GO-BP: Group I intron splicing | 1.75E-02 |
| GO-BP: RNA splicing, via transesterification reactions with guanosine as nucleophile | 1.75E-02 |
| GO-BP: RNA splicing, via transesterification reactions | 1.96E-02 |
| GO-BP: Response to antibiotic | 2.39E-02 |
| GO-BP: Organic acid phosphorylation | 2.40E-02 |
| GO-BP: Protein transport | 2.73E-02 |
| GO-BP: Homeostatic process | 2.73E-02 |
| GO-BP: Endomembrane system organization | 2.75E-02 |
| GO-BP: Tetrapyrrole metabolic process | 2.81E-02 |
| GO-BP: Peptide transport | 3.05E-02 |
| GO-BP: Amide transport | 3.28E-02 |
| GO-BP: RNA splicing | 3.31E-02 |
| GO-BP: Porphyrin-containing compound biosynthetic process | 3.31E-02 |
| GO-BP: Steroid biosynthetic process | 3.31E-02 |
| GO-BP: Steroid metabolic process | 3.48E-02 |
| GO-BP: Export from cell | 3.48E-02 |
| GO-BP: Intracellular protein transport | 3.48E-02 |
| GO-BP: Mitochondrial genome maintenance | 3.58E-02 |
| GO-BP: Ribonucleoprotein complex disassembly | 3.58E-02 |
| GO-BP: Spliceosomal complex disassembly | 3.58E-02 |
| GO-BP: Poly(hydroxyalkanoate) biosynthetic process | 3.58E-02 |
| GO-BP: Poly(hydroxyalkanoate) metabolic process | 3.58E-02 |
| GO-BP: Muscle organ development | 3.58E-02 |
| GO-BP: Visual perception | 3.58E-02 |
| GO-BP: Poly-hydroxybutyrate metabolic process | 3.58E-02 |
| GO-BP: Poly-hydroxybutyrate biosynthetic process | 3.58E-02 |
| GO-BP: Muscle structure development | 3.58E-02 |
| GO-BP: Sensory perception of light stimulus | 3.58E-02 |
| GO-BP: Porphyrin-containing compound metabolic process | 3.74E-02 |
| GO-BP: Tetrapyrrole biosynthetic process | 3.83E-02 |
| GO-BP: Endoplasmic reticulum inheritance | 3.90E-02 |
| GO-BP: Cell redox homeostasis | 4.02E-02 |

Table S8-1 Enriched GO Biological Process (GO-BP), and KEGG Pathway of positively selected genes in *Caulokaempferia coenobialis* (Cco) branch (continued)

| Term | *P*-value |
| --- | --- |
| GO-BP: Cell growth | 4.18E-02 |
| GO-BP: Heme biosynthetic process | 4.23E-02 |
| GO-BP: Nitrogen compound transport | 4.25E-02 |
| GO-BP: Histidine biosynthetic process | 4.47E-02 |
| GO-BP: Vesicle docking involved in exocytosis | 4.47E-02 |
| GO-BP: Exocytic process | 4.47E-02 |
| GO-BP: Glycosaminoglycan catabolic process | 4.47E-02 |
| GO-BP: Valyl-tRNA aminoacylation | 4.75E-02 |
| GO-BP: Peptidoglycan catabolic process | 4.75E-02 |
| GO-BP: Attachment of GPI anchor to protein | 4.75E-02 |
| GO-BP: rRNA modification | 4.76E-02 |
| GO-BP: Glycine metabolic process | 4.76E-02 |
| GO-BP: Organelle inheritance | 4.76E-02 |
| GO-BP: SRP-dependent cotranslational protein targeting to membrane | 4.92E-02 |
| GO-BP: Establishment of protein localization to endoplasmic reticulum | 4.92E-02 |
| GO-BP: Protein targeting to ER | 4.92E-02 |
| KEGG: Lectins | 1.93E-03 |
| KEGG: Folate biosynthesis | 8.68E-03 |
| KEGG: Various types of N-glycan biosynthesis | 1.30E-02 |
| KEGG: Glycosaminoglycan binding proteins | 1.56E-02 |
| KEGG: N-Glycan biosynthesis | 2.28E-02 |
| KEGG: Chaperones and folding catalysts | 2.70E-02 |
| KEGG: Transport | 4.01E-02 |

Table S8-2 Enriched GO Biological Process (GO-BP), and KEGG Pathway of positively selected genes in *Hornstedtia hainanensis* (Hhn) branch

| Term | *P*-value |
| --- | --- |
| GO-BP: Macromolecule localization | 1.27E-03 |
| GO-BP: Lipid transport | 1.36E-03 |
| GO-BP: Spindle pole body organization | 1.71E-03 |
| GO-BP: Spindle pole body duplication | 1.71E-03 |
| GO-BP: Dormancy process | 2.66E-03 |
| GO-BP: Lipid localization | 2.86E-03 |
| GO-BP: Regulation of cellular protein metabolic process | 2.93E-03 |
| GO-BP: Regulation of protein metabolic process | 3.42E-03 |
| GO-BP: Protein insertion into mitochondrial membrane | 3.52E-03 |
| GO-BP: Outer mitochondrial membrane organization | 3.52E-03 |
| GO-BP: Protein insertion into mitochondrial outer membrane | 3.52E-03 |
| GO-BP: Microtubule organizing center organization | 3.98E-03 |
| GO-BP: Attachment of GPI anchor to protein | 4.49E-03 |
| GO-BP: Establishment of protein localization to mitochondrial membrane | 5.57E-03 |
| GO-BP: Pyrimidine nucleoside metabolic process | 5.57E-03 |
| GO-BP: Protein folding | 5.74E-03 |
| GO-BP: Protein localization to spindle pole body | 8.05E-03 |
| GO-BP: Protein localization to microtubule organizing center | 8.05E-03 |
| GO-BP: Protein localization to microtubule cytoskeleton | 8.05E-03 |
| GO-BP: Protein localization to cytoskeleton | 8.05E-03 |
| GO-BP: Lipoprotein metabolic process | 9.24E-03 |
| GO-BP: Cell cycle | 1.02E-02 |
| GO-BP: Protein localization | 1.03E-02 |
| GO-BP: Viral capsid assembly | 1.08E-02 |
| GO-BP: Retrograde transport, endosome to Golgi | 1.25E-02 |
| GO-BP: Response to inorganic substance | 1.37E-02 |
| GO-BP: Group I intron splicing | 1.42E-02 |
| GO-BP: RNA splicing, via transesterification reactions with guanosine as nucleophile | 1.42E-02 |
| GO-BP: Peptide transport | 1.47E-02 |
| GO-BP: Amide transport | 1.53E-02 |
| GO-BP: Organic substance transport | 1.74E-02 |
| GO-BP: Nitrogen compound transport | 1.76E-02 |
| GO-BP: Establishment of protein localization | 1.77E-02 |
| GO-BP: Protein insertion into membrane | 1.78E-02 |
| GO-BP: Response to acid chemical | 1.88E-02 |
| GO-BP: Virion assembly | 2.17E-02 |

Table S8-2 Enriched GO Biological Process (GO-BP), and KEGG Pathway of positively selected genes in *Hornstedtia hainanensis* (Hhn) branch (continued)

| Term | *P*-value |
| --- | --- |
| GO-BP: Response to water | 2.18E-02 |
| GO-BP: Enamel mineralization | 2.29E-02 |
| GO-BP: Positive regulation of biomineral tissue development | 2.29E-02 |
| GO-BP: Regulation of tooth mineralization | 2.29E-02 |
| GO-BP: Regulation of enamel mineralization | 2.29E-02 |
| GO-BP: Positive regulation of tooth mineralization | 2.29E-02 |
| GO-BP: Positive regulation of enamel mineralization | 2.29E-02 |
| GO-BP: Amelogenesis | 2.29E-02 |
| GO-BP: Regulation of animal organ morphogenesis | 2.29E-02 |
| GO-BP: Regulation of odontogenesis | 2.29E-02 |
| GO-BP: Positive regulation of odontogenesis | 2.29E-02 |
| GO-BP: Regulation of odontogenesis of dentin-containing tooth | 2.29E-02 |
| GO-BP: Positive regulation of odontogenesis of dentin-containing tooth | 2.29E-02 |
| GO-BP: Positive regulation of animal organ morphogenesis | 2.29E-02 |
| GO-BP: Positive regulation of biomineralization | 2.29E-02 |
| GO-BP: Tooth mineralization | 2.29E-02 |
| GO-BP: Protein transport | 2.36E-02 |
| GO-BP: Cytosolic transport | 2.39E-02 |
| GO-BP: Cellular ion homeostasis | 2.54E-02 |
| GO-BP: Cellular metal ion homeostasis | 2.54E-02 |
| GO-BP: Cellular cation homeostasis | 2.54E-02 |
| GO-BP: RNA processing | 2.83E-02 |
| GO-BP: Cellular localization | 2.96E-02 |
| GO-BP: Metal ion homeostasis | 3.05E-02 |
| GO-BP: Calcium ion transport | 3.12E-02 |
| GO-BP: Divalent inorganic cation transport | 3.37E-02 |
| GO-BP: Urea transmembrane transport | 3.41E-02 |
| GO-BP: Obsolete mitochondrial respiratory chain complex IV biogenesis | 3.41E-02 |
| GO-BP: One-carbon compound transport | 3.41E-02 |
| GO-BP: Urea transport | 3.41E-02 |
| GO-BP: Odontogenesis of dentin-containing tooth | 3.41E-02 |
| GO-BP: Odontogenesis | 3.41E-02 |
| GO-BP: Carbohydrate derivative catabolic process | 3.52E-02 |
| GO-BP: Mitochondrial membrane organization | 3.57E-02 |
| GO-BP: Regulation of translation | 3.66E-02 |
| GO-BP: Regulation of cellular amide metabolic process | 3.66E-02 |
| GO-BP: ncRNA processing | 3.73E-02 |
| GO-BP: Cellular chemical homeostasis | 3.75E-02 |

Table S8-2 Enriched GO Biological Process (GO-BP), and KEGG Pathway of positively selected genes in *Hornstedtia hainanensis* (Hhn) branch (continued)

| Term | *P*-value |
| --- | --- |
| GO-BP: Endosomal transport | 3.83E-02 |
| GO-BP: Lysine catabolic process | 3.83E-02 |
| GO-BP: Aspartate family amino acid catabolic process | 3.83E-02 |
| GO-BP: Cellular macromolecule localization | 3.99E-02 |
| GO-BP: Cellular protein localization | 3.99E-02 |
| GO-BP: Posttranscriptional regulation of gene expression | 4.04E-02 |
| GO-BP: Regulation of protein modification process | 4.05E-02 |
| GO-BP: SOS response | 4.36E-02 |
| GO-BP: Intracellular transport | 4.41E-02 |
| GO-BP: Cytidine catabolic process | 4.52E-02 |
| GO-BP: Regulation of oxidoreductase activity | 4.52E-02 |
| GO-BP: Negative regulation of oxidoreductase activity | 4.52E-02 |
| GO-BP: Regulation of D-amino-acid oxidase activity | 4.52E-02 |
| GO-BP: Negative regulation of D-amino-acid oxidase activity | 4.52E-02 |
| GO-BP: Biomineral tissue development | 4.52E-02 |
| GO-BP: Regulation of biomineral tissue development | 4.52E-02 |
| GO-BP: Maintenance of DNA repeat elements | 4.52E-02 |
| GO-BP: Maintenance of CRISPR repeat elements | 4.52E-02 |
| GO-BP: Pyrimidine-containing compound catabolic process | 4.52E-02 |
| GO-BP: Nucleoside catabolic process | 4.52E-02 |
| GO-BP: Cytidine metabolic process | 4.52E-02 |
| GO-BP: Ectoine biosynthetic process | 4.52E-02 |
| GO-BP: Pyrimidine ribonucleoside catabolic process | 4.52E-02 |
| GO-BP: Pyrimidine nucleoside catabolic process | 4.52E-02 |
| GO-BP: Glycosyl compound catabolic process | 4.52E-02 |
| GO-BP: Chorismate metabolic process | 4.52E-02 |
| GO-BP: Response to host immune response | 4.52E-02 |
| GO-BP: Ectoine metabolic process | 4.52E-02 |
| GO-BP: Ribonucleoside catabolic process | 4.52E-02 |
| GO-BP: Regulation of biomineralization | 4.52E-02 |
| GO-BP: Biomineralization | 4.52E-02 |
| GO-BP: Animal organ morphogenesis | 4.52E-02 |
| GO-BP: Cytidine deamination | 4.52E-02 |
| GO-BP: Evasion of host immune response | 4.52E-02 |
| GO-BP: Nucleobase-containing small molecule catabolic process | 4.52E-02 |
| GO-BP: Fucose metabolic process | 4.52E-02 |
| GO-BP: Establishment of localization in cell | 4.62E-02 |
| GO-BP: rRNA processing | 4.63E-02 |

Table S8-2 Enriched GO Biological Process (GO-BP), and KEGG Pathway of positively selected genes in *Hornstedtia hainanensis* (Hhn) branch (continued)

| Term | *P*-value |
| --- | --- |
| GO-BP: RRNA metabolic process | 4.63E-02 |
| GO-BP: Protein import | 4.70E-02 |
| GO-BP: Cytoskeleton organization | 4.85E-02 |
| KEGG: Valine, leucine and isoleucine degradation | 7.73E-03 |
| KEGG: Chromosome and associated proteins | 1.67E-02 |
| KEGG: Folate biosynthesis | 1.84E-02 |
| KEGG: Cell growth | 2.32E-02 |
| KEGG: Chaperones and folding catalysts | 2.35E-02 |
| KEGG: Ribosome biogenesis in eukaryotes | 3.19E-02 |
| KEGG: Caffeine metabolism | 3.46E-02 |
| KEGG: Glycerolipid metabolism | 4.09E-02 |
| KEGG: Ubiquitin mediated proteolysis | 4.10E-02 |
| KEGG: Propanoate metabolism | 4.18E-02 |

Table S9 Candidates genes of *Caulokaempferia coenobialis* and *Hornstedtia hainanensis* involved in mucilage-like or gum-like pollen coat formation

Table S9-1 Candidates' genes of *Caulokaempferia coenobialis* involved in mucilage-like pollen coat formation

| Unigene ID | Arabidopsis accession | Function | TAIR Description |
| --- | --- | --- | --- |
| c16484_g2 | AT1G23090 | transport | Encodes AST91 mRNA for sulfate transporter. |
| c41539_g1 | AT4G27940 | transport | manganese tracking factor for mitochondrial SOD2;(source:Araport11) |
| c74617_g1 | AT3G53510 | transport | Belongs to a clade of five Arabidopsis thaliana ABCG half-transporters that are required for synthesis of an effective suberin barrier in roots and seed coats (ABCG2, ABCG6, and ABCG20) and for synthesis of an intact pollen wall (ABCG1 and ABCG16). Phloem-expressed and plasma membrane-localized jasmonate transporter which together with JAT3 and GLR3.3 involved in regulating long-distance translocation of JA, which is important for driving the loading, translocation of JA in the phloem pathway by a self-propagation mode, contributing to wound-induced systemic response/resistance. |
| c8111_g1 | AT1G14140 | transport | Mitochondrial substrate carrier family protein;(source: Araport11) |
| c26296_g1 | AT1G47830 | transport | SNARE-like superfamily protein;(source:Araport11) |
| c40176_g1 | AT1G71820 | transport | Encodes a member of the exocyst complex gene family. The exocyst is a protein complex involved in tethering vesicles to the plasma membrane during regulated or polarized secretion. |
| c65670_g1 | AT2G28520 | transport | Vacuolar proton ATPase subunit VHA-a isoform 1. Localized in the trans-Golgi network. The mRNA is cell-to-cell mobile. |
| c51468_g1 | AT2G44100 | transport | GDP dissociation inhibitor involved in vesicular membrane traffic |
| c66046_g1 | AT3G04090 | transport | Belongs to a family of plant aquaporins. Similar to yeast and radish aquaporins. Located on ER. |
| c56768_g1 | AT3G07950 | transport | rhomboid protein-like protein;(source:Araport11) |
| c56666_g1 | AT3G43790 | transport | zinc induced facilitator-like 2;(source:Araport11) |
| c62966_g1 | AT3G58970 | transport | Transmembrane magnesium transporter. One of nine family members. |

Table S9-1 Candidates' genes of *Caulokaempferia coenobialis* involved in mucilage-like pollen coat formation (continued)

| Unigene ID | Arabidopsis accession | Function | TAIR Description |
| --- | --- | --- | --- |
| c73851_g1 | AT3G02850 | transport | Encodes SKOR, a member of Shaker family potassium ion (K+) channel. This family includes five groups based on phylogenetic analysis (FEBS Letters (2007) 581: 2357): I (inward rectifying channel): AKT1 (AT2G26650), AKT5 (AT4G32500) and SPIK (also known as AKT6, AT2G25600); II (inward rectifying channel): KAT1 (AT5G46240) and KAT2 (AT4G18290); III (weakly inward rectifying channel): AKT2 (AT4G22200); IV (regulatory subunit involved in inwardly rectifying conductance formation): KAT3 (also known as AtKC1, AT4G32650); V (outward rectifying channel): SKOR (AT3G02850) and GORK (AT5G37500). Mediates the delivery of K+ from stelar cells to the xylem in the roots towards the shoot. mRNA accumulation is modulated by abscisic acid. K+ gating activity is modulated by external and internal K+. Involved in response to low potassium. |
| c65645_g1 | AT3G45650 | transport | Encodes a nitrate efflux transporter NAXT1 (for NITRATE EXCRETION TRANSPORTER1). Localized to the plasma membrane. NAXT1 belongs to a subclass of seven NAXT members from the large NITRATE TRANSPORTER1/PEPTIDE TRANSPORTER family and is mainly expressed in the cortex of mature roots. |
| c59627_g1 | AT3G47700 | transport | Involved in transportation of seed storage proteins from the ER to the vacuole. Mutant seed cell accumulates the precursors of 12S globulin and 2S albumin instead of the vacuolar-located mature proteins. Member of MAG2 complex, involved in the development of vegetative organs. |
| c60959_g1 | AT4G12780 | transport | Negative regulation of growth and endocytosis, most likely as a result of inhibition of the recruitment of clathrin to endocytic pits. Overexpression inhibits recruitment of clathrin resulting in negative regulation of endocytosis and developmental arrest. |
| c51826_g2 | AT4G15080 | transport | DHHC-type zinc finger family protein;(source: Araport11) |
| c62982_g1 | AT5G25400 | transport | Nucleotide-sugar transporter family protein;(source: Araport11) |

Table S9-1 Candidates' genes of *Caulokaempferia coenobialis* involved in mucilage-like pollen coat formation (continued)

| Unigene ID | Arabidopsis accession | Function | TAIR Description |
| --- | --- | --- | --- |
| c58779_g1 | AT5G03555 | transport | Encodes PLUTO (plastidic nucleobase transporter), a member of the Nucleobase: Cation-Symporter1 protein family, capable of transporting purine and pyrimidine nucleobases. |
| c64096_g1 | AT5G19380 | transport | Encodes one of the CRT-Like transporters (CLT1/AT5G19380, CLT2/AT4G24460, CLT3/AT5G12170). Required for glutathione homeostasis and stress responses. Mutants lacking these transporters are heavy metal-sensitive, glutathione(GSH)-deficient, and hypersensitive to Phytophthora infection. |
| c50704_g1 | AT5G47480 | transport | RGPR-related protein; SEC16A homolog. Part of endomembrane trafficking system. |
| c48162_g2 | AT5G64290 | transport | dicarboxylate transport 2.1;(source:Araport11) |
| c61087_g1 | AT1G80680 | transport | Mutant has early-flowering phenotype, encodes a putative nucleoporin. Required for the activation of downstream defense pathways by the snc1 mutation. Involved in basal resistance against bacterial pathogens. |
| c45359_g1 | AT3G21260 | transport | Glycolipid transfer protein (GLTP) family protein;(source: Araport11) |
| c45125_g1 | ATMG00580 | transport | NADH dehydrogenase subunit 4 |
| c101448_g1 | AT5G35080 | transport | Encodes a protein involved in the endoplasmic reticulum-associated degradation of glycoproteins. |
| c61480_g1 | AT5G60960 | transport | Encodes PNM1 (for PPR protein localized to the nucleus and mitochondria 1), a PPR protein that is dual localized to mitochondria and nuclei. Loss of PNM1 function in mitochondria, but not in nuclei, is lethal for the embryo. In mitochondria, it is associated with polysomes and may play a role in translation. |
| c45105_g1 | AT4G28500 | regulation of secondary cell wall biogenesis | NAC domain containing protein 73;(source: Araport11) |

Table S9-1 Candidates' genes of *Caulokaempferia coenobialis* involved in mucilage-like pollen coat formation (continued)

| Unigene ID | Arabidopsis accession | Function | TAIR Description |
| --- | --- | --- | --- |
| c56337_g1 | AT1G32200 | Lipid metabolism | Encodes a chloroplast glycerol-3-phosphate acyltransferase. Involved in the biosynthesis of chloroplast phosphatidylglycerol. |
| c66616_g1 | AT1G76150 | Lipid metabolism | Encodes a monofunctional enoyl-CoA hydratase 2, involved in the degradation of even cis-unsaturated fatty acids, gene expression is enhanced during the first 2 days of germination, as well as in senescent leaves. |
| c51820_g1 | AT3G48460 | Lipid metabolism | GDSL-motif esterase/acyltransferase/lipase. Enzyme group with broad substrate specificity that may catalyze acyltransfer or hydrolase reactions with lipid and non-lipid substrates. |
| c58829_g2 | AT3G54610 | Lipid metabolism | Encodes a histone acetyltransferase that plays a role in the determination of the embryonic root-shoot axis. It is also required to regulate the floral meristem activity by modulating the extent of expression of WUS and AG. In addition, it is involved in stem cuticular wax accumulation by modulating CER3 expression via H3K9/14 acetylation. In other eukaryotes, this protein is recruited to specific promoters by DNA binding transcription factors and is thought to promote transcription by acetylating the N-terminal tail of histone H3. The enzyme has indeed been shown to catalyse primarily the acetylation of H3 histone with only traces of H4 and H2A/B being acetylated. Non-acetylated H3 peptide or an H3 peptide that had been previously acetylated on K9 both serve as excellent substrates for HAG1-catalyzed acetylation. However, prior acetylation of H3 lysine 14 blocks radioactive acetylation of the peptide by HAG1. HAG1 is specific for histone H3 lysine 14. |
| c60077_g1 | AT5G23670 | Lipid metabolism | Encodes the LCB2 subunit of serine palmitoyltransferase, an enzyme involved in sphingosine biosynthesis. The protein is localized to the endoplasmic reticulum. |
| c90986_g1 | AT4G00360 | Lipid metabolism | Encodes a member of the CYP86A subfamily of cytochrome p450 genes. Expressed at moderate levels in flowers, leaves, roots and stems. |

Table S9-1 Candidates' genes of *Caulokaempferia coenobialis* involved in mucilage-like pollen coat formation (continued)

| Unigene ID | Arabidopsis accession | Function | TAIR Description |
| --- | --- | --- | --- |
| c50207_g1 | AT3G14110 | Lipid metabolism | Encodes a novel coiled-coil, TPR domain containing protein that is localized to the chloroplast membrane and is involved in chlorophyll biosynthesis. Mutants accumulate protochlorophyllide, an intermediate in the chlorophyll biosynthesis pathway, in dark and release singlet oxygen in plastids in a controlled and non-invasive manner upon a dark/light shift. |
| c61926_g2 | AT3G26840 | Lipid metabolism | Encodes a protein with phytyl ester synthesis and diacylglycerol acyltransferase activities that is involved in the deposition of free phytol and free fatty acids in the form of phytyl esters in chloroplasts, a process involved in maintaining the integrity of the photosynthetic membrane during abiotic stress and senescence. |
| c49913_g1 | AT4G30340 | Lipid metabolism | encodes a diacylglycerol kinase. Applying a specific diacylglycerol kinase inhibitor to the growth media resulted in reduced root elongation and plant growth. Gene is expressed throughout the plant but is strongest in flowers and young seedlings. |
| c64993_g1 | AT1G08550 | Lipid metabolism | Violaxanthin deepoxidase involved in xanthophyll cycle. Two major consequences of the npq1 mutation are the absence of zeaxanthin formation in strong light and the partial inhibition of the quenching of singlet excited chlorophylls in the photosystem II light-harvesting complex |
| c47747_g1 | AT1G47290 | Lipid metabolism | Encodes an enzyme with 3β-hydroxysteroid dehydrogenase/C4-decarboxylase activity *in vitro*. The activity of the enzyme was determined using microsomal extracts of yeast overexpressing the *Arabidopsis* gene. Cytosolic fractions failed to be associated to the activity, leading to the speculation that the enzyme is membrane-bound. |
| c8256_g1 | AT1G65520 | Lipid metabolism | encodes a peroxisomal delta3, delta2-enoyl CoA isomerase, involved in unsaturated fatty acid degradation |

Table S9-1 Candidates' genes of *Caulokaempferia coenobialis* involved in mucilage-like pollen coat formation (continued)

| Unigene ID | Arabidopsis accession | Function | TAIR Description |
| --- | --- | --- | --- |
| c65119_g1 | AT5G03730 | Lipid metabolism | Homologous to the RAF family of serine/threonine protein kinases. Negative regulator in the ethylene signal transduction pathway. Interacts with the putative ethylene receptors ETR1 and ERS. Constitutively expressed. |
| c30625_g1 | AT5G57030 | Lipid metabolism | Lutein-deficient 2 (LUT2) required for lutein biosynthesis, member of the xanthophyll class of carotenoids. Encodes lycopene epsilon cyclase |
| c15269_g1 | AT1G01200 | cell wall biogenesis | RAB GTPase homolog A3;(source:Araport11) |
| c55551_g1 | AT1G15690 | Carbohydrate metabolism | Encodes a H(+)-translocating (pyrophosphate-energized) inorganic pyrophosphatase (H(+)-PPase; EC 3.6.1.1) located in the vacuolar membrane. Expression is found in all tissues examined, including meristems and floral organ primordium. Expression is particularly enhanced in pollen, and is repressed by light. Over expression and loss of function phenotypes suggest AVP1 is involved in regulation of apoplastic pH and auxin transport. The effect on auxin transport likely involves effects of extracellular pH on subcellular localization of auxin efflux carriers such as PIN1. The mRNA is cell-to-cell mobile. |
| c62854_g1 | AT1G16900 | Carbohydrate metabolism | Encodes the Arabidopsis ortholog of the yeast/human ALG9 catalyzing the luminal addition of two alpha-1,2 Man residues in assembling Glc3Man9GlcNAc2. |
| c55118_g1 | AT1G27440 | Carbohydrate metabolism | IRX10 was identified as MUCI69 in a reverse genetic screen for MUCILAGE-RELATED genes. Mutations in this gene did not disrupt mucilage properties, likely due to the presence of the functionally redundant IRX10-L. |
| c31793_g1 | AT1G32860 | Carbohydrate metabolism | Glycosyl hydrolase superfamily protein;(source: Araport11) |
| c64200_g1 | AT1G51590 | Carbohydrate metabolism | Encodes an alpha-mannosidase I enzyme responsible for N-glycan maturation. |

Table S9-1 Candidates' genes of *Caulokaempferia coenobialis* involved in mucilage-like pollen coat formation (continued)

| Unigene ID | Arabidopsis accession | Function | TAIR Description |
| --- | --- | --- | --- |
| c63290_g1 | AT2G20370 | Carbohydrate metabolism | Encodes a xyloglucan galactosyltransferase located in the membrane of Golgi stacks that is involved in the biosynthesis of fucose. It is also involved in endomembrane organization. It is suggested that it is a dual-function protein that is responsible for actin organization and the synthesis of cell wall materials. The mRNA is cell-to-cell mobile. |
| c57885_g1 | AT3G23920 | Carbohydrate metabolism | Encodes a chloroplast beta-amylase. Is necessary for leaf starch breakdown in the absence of BAM3.Activity of BAM1 increases 4 days after osmotic stress. BAM1 has a higher temperature optimum than BAM3 (PMID:25293962). |
| c28162_g1 | AT4G13210 | Carbohydrate metabolism | Pectin lyase-like superfamily protein;(source: Araport11) |
| c65127_g1 | AT5G15630 | Carbohydrate metabolism | Encodes a member of the COBRA family, similar to phytochelatin synthetase. Involved in secondary cell wall biosynthesis. Mutants make smaller plants with reduced levels of cellulose and cell wall sugars. |
| c65524_g1 | AT5G19690 | Carbohydrate metabolism | encodes an oligosaccharyl transferase involved response to high salt. Mutants are hypersensitive to high salt conditions The mRNA is cell-to-cell mobile. |
| c36171_g1 | AT5G24300 | Carbohydrate metabolism | SSI is a plastidial enzyme and crucial for the synthesis of normal amylopectin in the leaves of Arabidopsis. The absence of SSI results in a deficiency in the number of shorter glucans which in turn affect the formation and connection of the amylopectin clusters in starch. |
| c54712_g1 | AT5G51970 | Carbohydrate metabolism | Encodes a sorbitol dehydrogenase that exhibits greatest oxidative activity with sorbitol, ribitol and xylitol as substrates. SDH can be thiolated in vitro. |
| c57141_g1 | AT5G56360 | Carbohydrate metabolism | Encodes PSL4, beta-subunit of endoplasmic reticulum-resident glucosidase II, which is essential for stable accumulation and quality control of the elf18 receptor EFR but not the flg22 receptor FLS2. The mRNA is cell-to-cell mobile. |
| c47196_g1 | AT3G06850 | Carbohydrate metabolism | dihydrolipoamide branched chain acyltransferase |

Table S9-1 Candidates' genes of *Caulokaempferia coenobialis* involved in mucilage-like pollen coat formation (continued)

| Unigene ID | Arabidopsis accession | Function | TAIR Description |
| --- | --- | --- | --- |
| c57063_g1 | AT4G26910 | Carbohydrate metabolism | Encodes the E2 subunit of the 2-oxoglutarate dehydrogenase. |
| c37168_g1 | AT1G06550 | Carbohydrate metabolism | ATP-dependent caseinolytic (Clp) protease/crotonase family protein;(source:Araport11) |

Table S9-2 Candidates' genes of *Hornstedtia hainanensis* involved in mucilage-like pollen coat formation (continued)

| Unigene ID | Arabidopsis accession | Function | TAIR Description |
| --- | --- | --- | --- |
| c93811_g1 | AT5G12110 | Transcription Factors | elongation factor 1-beta 1;(source:Araport11) |
| c19830_g2 | AT4G02700 | Transport | sulfate transporter 3;(source:Araport11) |
| c67343_g1 | AT1G12520 | transport | Copper-zinc superoxide dismutase copper chaperone (delivers copper to the Cu-Zn superoxide dismutase). Localized to the chloroplast. Expressed in roots and shoots. Up-regulated in response to copper and senescence. The AtACC activates all three CuZnSOD activities located in three different subcellular compartments. Contains three domains, central, ATX-1 like and C-terminal. ATX-1 like domain essential for the copper chaperone function of AtCCS in planta. |
| c14447_g1 | AT1G27950 | transport | Encodes LTPG1, a lipid transfer protein with a predicted GPI (glycosylphosphatidylinositol)-anchor domain. Localized in the plasma membrane. Disruption of the LTPG1 gene causes alterations of cuticular lipid composition, but no significant changes on total wax and cutin monomer loads are seen. The mRNA is cell-to-cell mobile. |
| c55055_g1 | AT1G64780 | transport | encodes an ammonium transporter protein believed to act as a high affinity transporter. It is expressed in the root, primarily in endodermal and cortical cells, and contributes to ammonium uptake in the root. |
| c66084_g1 | AT1G65410 | transport | Encodes a member of NAP subfamily of transporters. Mutations in this gene suppress the low temperature-induced phenotype of Arabidopsis tocopherol-deficient mutant vte2. |
| c53668_g1 | AT2G32900 | transport | Homologous to Drosophila ZW10, a centromere/kinetochore protein involved in chromosome segregation. Member of MAG2 complex on the ER that is responsible for efficient transport of seed storage proteins, functions in protein transport between the ER and Golgi apparatus, contain a Zeste? White 10 (ZW10) domain and a Sec39 domain. Required for proper maturation of seed storage proteins. |

Table S9-2 Candidates' genes of *Hornstedtia hainanensis* involved in mucilage-like pollen coat formation (continued)

| Unigene ID | Arabidopsis accession | Function | TAIR Description |
| --- | --- | --- | --- |
| c45170_g2 | AT3G02850 | transport | Encodes SKOR, a member of Shaker family potassium ion (K+) channel. This family includes five groups based on phylogenetic analysis (FEBS Letters (2007) 581: 2357): I (inward rectifying channel): AKT1 (AT2G26650), AKT5 (AT4G32500) and SPIK (also known as AKT6, AT2G25600); II (inward rectifying channel): KAT1 (AT5G46240) and KAT2 (AT4G18290); III (weakly inward rectifying channel): AKT2 (AT4G22200); IV (regulatory subunit involved in inwardly rectifying conductance formation): KAT3 (also known as AtKC1, AT4G32650); V (outward rectifying channel): SKOR (AT3G02850) and GORK (AT5G37500). Mediates the delivery of K+ from stelar cells to the xylem in the roots towards the shoot. mRNA accumulation is modulated by abscisic acid. K+ gating activity is modulated by external and internal K+. Involved in response to low potassium. |
| c46423_g1 | AT3G05030 | transport | Encodes a vacuolar K+/H+ exchanger essential for active K+ uptake at the tonoplast and involved in regulating stomatal closure. |
| c44623_g1 | AT3G51780 | transport | A member of Arabidopsis BAG (Bcl-2-associated athanogene) proteins, plant homologs of mammalian regulators of apoptosis. BD domain of ATBAG4 had highest similarity to human DB domain of BAG protein. Plant BAG proteins are multi-functional and remarkably similar to their animal counterparts, as they regulate apoptotic-like processes ranging from pathogen attack, to abiotic stress, to plant development. |
| c67071_g2 | AT3G61710 | transport | Encodes autophagy protein 6 (ATG6), required for pollen germination and plant development. |
| c43705_g2 | AT4G13590 | transport | Chloroplast manganese transporter required for chloroplast manganese homeostasis and photosynthetic function. |

Table S9-2 Candidates' genes of *Hornstedtia hainanensis* involved in mucilage-like pollen coat formation (continued)

| Unigene ID | Arabidopsis accession | Function | TAIR Description |
| --- | --- | --- | --- |
| c52469_g1 | AT4G27540 | transport | prenylated RAB acceptor 1.H;(source:Araport11) |
| c107693_g1 | AT4G38580 | transport | putative farnesylated protein (At4g38580) mRNA, complete |
| c22991_g1 | AT5G01500 | transport | encodes an ATP/ADP carrier that is located to the thylakoid membrane involved in providing ATP during thylakoid biogenesis and turnover The mRNA is cell-to-cell mobile. |
| c58455_g1 | AT5G21930 | transport | P-Type ATPase, mediates copper transport to chloroplast thylakoid lumen. Required for accumulation of copper-containing plastocyanin in the thylakoid lumen and for effective photosynthetic electron transport |
| c65944_g1 | AT5G25400 | transport | Nucleotide-sugar transporter family protein;(source: Araport11) |
| c57367_g1 | AT5G50300 | transport | Encodes a homolog of the adenine-guanine-hypoxanthine transporter AzgA of Aspergillus nidulans. Function as a plant adenine-guanine transporter. Two closely related genes exist in Arabidopsis: AT3G10960 (Azg1) and AT5G50300 (Azg2). |
| c57333_g1 | AT5G54440 | transport | Encodes a putative TRAPP II tethering factor required for cell plate assembly during cytokinesis. Part of multi-protein complex, acting as guanine nucleotide exchange factors (GEFs) and possibly as tethers, regulating intracellular trafficking. |
| c65072_g1 | AT5G62730 | transport | Major facilitator superfamily protein;(source: Araport11) |
| c63709_g2 | ATMG00580 | transport | NADH dehydrogenase subunit 4 |
| c62395_g7 | AT3G21260 | transport | Glycolipid transfer protein (GLTP) family protein;(source: Araport11) |
| c4693_g1 | AT5G42960 | transport | outer envelope pore 24B-like protein;(source:Araport11) |
| c95712_g1 | AT2G15570 | transport | chloroplast protein similar to prokaryotic thioredoxin. |
| c58936_g1 | AT2G05120 | transport | Nucleoporin, Nup133/Nup155-like protein;(source:Araport11) |

Table S9-2 Candidates' genes of *Hornstedtia hainanensis* involved in mucilage-like pollen coat formation (continued)

| Unigene ID | Arabidopsis accession | Function | TAIR Description |
| --- | --- | --- | --- |
| c57905_g1 | AT5G11710 | transport | EPSIN1 plays an important role in the vacuolar trafficking of soluble proteins at the trans-Golgi network via its interaction with gamma-ADR, VTI11, VSR1, and clathrin. Associated with actin filaments and with the Golgi complex. Expressed in most tissues. The mRNA is cell-to-cell mobile. |
| c65581_g2 | AT5G40930 | transport | Form of TOM20, which is a component of the TOM complex involved in transport of nuclear-encoded mitochondrial proteins |
| c7864_g1 | AT5G55220 | transport | Contains with HP22 a protein that is related to the bacterial trigger factor chaperone. Plants depleted of either HP22 or HP65b or even both were increasingly delayed in leaf senescence and retained much longer stromal chloroplast constituents than wild-type plants. |
| c39068_g1 | AT1G12500 | transport | Nucleotide-sugar transporter family protein;(source: Araport11) |
| c45094_g1 | AT1G31470 | transport | Major facilitator superfamily protein;(source: Araport11) |
| c120199_g1 | AT1G64550 | transport | Encodes a member of GCN subfamily. Predicted to be involved in stress-associated protein translation control. The mutant is affected in MAMP ((microbe-associated molecular patterns)-induced stomatal closure, but not other MAMP-induced responses in the leaves. Arabidopsis has five ABCF proteins, which are all closely related by sequence to yeast GCN20. None of these five are individually required for GCN2 kinase activity. |
| c57797_g1 | AT2G29050 | transport | RHOMBOID-like 1;(source:Araport11) |
| c120042_g1 | AT4G37680 | transport | heptahelical transmembrane protein HHP4 |
| c87476_g1 | AT5G05820 | transport | Nucleotide-sugar transporter family protein;(source: Araport11) |
| c18015_g1 | AT1G31190 | lipid /carbohydrate metabolic process | Encodes a myo-inositol monophosphatase IMPL1 (myo-Inositol monophosphatase like 1). |

Table S9-2 Candidates' genes of *Hornstedtia hainanensis* involved in mucilage-like pollen coat formation (continued)

| Unigene ID | Arabidopsis accession | Function | TAIR Description |
| --- | --- | --- | --- |
| c66564_g3 | AT4G13830 | lipid /carbohydrate metabolic process | DnaJ-like protein (J20); nuclear gene |
| c139528_g1 | AT1G68530 | Lipid metabolism | Encodes KCS6, a member of the 3-ketoacyl-CoA synthase family involved in the biosynthesis of VLCFA (very long chain fatty acids). |
| c57004_g1 | AT1G74320 | Lipid metabolism | encodes a choline kinase, whose expression is induced by high salt and mannitol. |
| c131789_g1 | AT2G26710 | Lipid metabolism | Encodes a member of the cytochrome p450 family that serves as a control point between multiple photoreceptor systems and brassinosteroid signal transduction. Involved in brassinolide metabolism. Mediates response to a variety of light signals including hypocotyl elongation and cotyledon expansion. |
| c60200_g1 | AT2G38010 | Lipid metabolism | Neutral/alkaline non-lysosomal ceramidase;(source: Araport11) |
| c12847_g1 | AT2G38110 | Lipid metabolism | bifunctional sn-glycerol-3-phosphate 2-O-acyltransferase/phosphatase. Involved in cutin assembly. |
| c68385_g1 | AT3G04870 | Lipid metabolism | Involved in the biosynthesis of carotenes and xanthophylls, reduces zeta-carotene to lycopene. |
| c107104_g1 | AT4G25050 | Lipid metabolism | encodes an acyl carrier protein predominantly expressed in leaves. Gene expression is upregulated by light. |

Table S9-2 Candidates' genes of *Hornstedtia hainanensis* involved in mucilage-like pollen coat formation (continued)

| Unigene ID | Arabidopsis accession | Function | TAIR Description |
| --- | --- | --- | --- |
| c43234_g1 | AT4G36810 | Lipid metabolism | Encodes a protein with geranylgeranyl pyrophosphate synthase activity involved in isoprenoid biosynthesis. The enzyme appears to be targeted to the chloroplast in epidermal cells and guard cells of leaves, and in etioplasts in roots. The mRNA is cell-to-cell mobile. |
| c80415_g1 | AT3G06810 | Lipid metabolism | Encodes a protein with similarity to acyl-CoA dehydrogenases. Mutations in IBR3 render plants resistant to indole-3-butryic acid, a putative storage form of the biologically active auxin IAA (indole-3-acetic acid). IBR3 is hypothesized to carry out the second step in a β-oxidation-like process of IBA metabolism in Arabidopsis. Though its subcellular location has not been determined, IBR3 has a peroxisomal targeting sequence and two other putative IBA metabolic enzymes (IBR1 and IBR10) can be found in this organelle. No specific enzymatic activity has been documented for IBR3, but double mutant analyses with CHY1 argue against a role for IBR3 in general fatty acid β-oxidation. The mRNA is cell-to-cell mobile. |
| c15019_g1 | AT5G06090 | lipid metabolism | putative sn-glycerol-3-phosphate 2-O-acyltransferase |
| c48344_g1 | AT5G23190 | Lipid metabolism | cytochrome P450 CYP86B1, nuclear gene for chloroplast product. CYP86B1 is a very long chain fatty acid hydroxylase specifically involved in polyester monomer biosynthesis during the course of plant development. |
| c54823_g2 | AT5G57690 | Lipid metabolism | Involved in nitric oxide-dependent pollen tube guidance and fertilization. |

Table S9-2 Candidates' genes of *Hornstedtia hainanensis* involved in mucilage-like pollen coat formation (continued)

| Unigene ID | Arabidopsis accession | Function | TAIR Description |
| --- | --- | --- | --- |
| c73570_g1 | AT1G32640 | flavonoid metabolic process/Transcription factors | Encodes a MYC-related transcriptional activator with a typical DNA binding domain of a basic helix-loop-helix leucine zipper motif. Binds to an extended G-Box promoter motif and interacts with Jasmonate ZIM-domain proteins. MYC2 interacts with EIN3 and EIL1 to repress hook curvature and resistance to Botrytis cinera.Its transcription is induced by dehydration stress, ABA treatment and blue light via CRY1. Negative regulator of blue light-mediated photomorphogenic growth and blue and far-red-light-regulated gene expression. Positive regulator of lateral root formation. Regulates diverse JA-dependent functions. Negatively regulates Trp metabolism and biosynthesis of Trp-derived secondary metabolites. Positively regulates flavonoid biosynthesis, resistance to insects, and response to oxidative stress. Regulates other transcription factors, and negatively regulates its own expression. For example, it binds to and regulates the expression of NST1. Its stability is modulated by PUB10 through polyubiquitination. |
| c46032_g1 | AT3G07020 | Carbohydrate metabolism/Lipid metabolism | encodes a 3beta-hydroxy sterol UDP-glucosyltransferase. ugt80a2 mutant plants have reduced steryl glycoside and acyl steryl glycoside levels and reduced seed size. ugt80a2/b1 double mutants have normal levels of celluose and normal cold stress tolerance. |
| c48422_g1 | AT2G14170 | Carbohydrate metabolism | Arabidopsis thaliana methylmalonate-semialdehyde dehydrogenase |
| c64992_g3 | AT2G26440 | Carbohydrate metabolism | Plant invertase/pectin methylesterase inhibitor superfamily;(source: Araport11) |
| c10091_g1 | AT3G01510 | Carbohydrate metabolism | Encodes a putative phosphatase, LSF1, required for normal starch turnover in leaves. |

Table S9-2 Candidates' genes of *Hornstedtia hainanensis* involved in mucilage-like pollen coat formation (continued)

| Unigene ID | Arabidopsis accession | Function | TAIR Description |
| --- | --- | --- | --- |
| c59656_g2 | AT2G35100 | Carbohydrate metabolism | Putative glycosyltransferase, similar to other CAZy Family 47 proteins. The protein is predicted to be a type 2 membrane protein with a signal anchor and is predicted to be targeted to the secretory pathway and to have a single transmembrane helix near the N terminus; hence, the protein has the features expected for a type II membrane protein targeted to the Golgi vesicles. The gene was shown to be expressed in all tissues but particularly in vascular tissues of leaves and stems. |
| c52653_g1 | AT3G02210 | Carbohydrate metabolism | COBRA-like protein 1 precursor;(source:Araport11) |
| c39127_g1 | AT3G06850 | Carbohydrate metabolism | dihydrolipoamide branched chain acyltransferase |
| c64094_g4 | AT3G23920 | Carbohydrate metabolism | Encodes a chloroplast beta-amylase. Is necessary for leaf starch breakdown in the absence of BAM3.Activity of BAM1 increases 4 days after osmotic stress. BAM1 has a higher temperature optimum than BAM3 (PMID:25293962). |
| c58035_g3 | AT3G55260 | Carbohydrate metabolism | Encodes a protein with β-hexosaminidase activity (the enzyme is active with p-nitrophenyl-β-N-acetylglucosaminide as substrate but displayed only a minor activity toward p-nitrophenyl-β-N-acetylgalactosaminide). The enzyme displays no distinct preference for a specific terminal GlcNAc residue and indeed cleaved the asialoagalactodabsylglycopeptide GnGn to a mixture of products. |
| c60548_g1 | AT4G33330 | Carbohydrate metabolism | Encodes a glucuronyltransferase responsible for the addition of GlcA residues onto xylan and for secondary wall deposition. |
| c66674_g1 | AT5G19690 | Carbohydrate metabolism | encodes an oligosaccharyl transferase involved response to high salt. Mutants are hypersensitive to high salt conditions The mRNA is cell-to-cell mobile. |

Table S9-2 Candidates' genes of *Hornstedtia hainanensis* involved in mucilage-like pollen coat formation (continued)

| Unigene ID | Arabidopsis accession | Function | TAIR Description |
| --- | --- | --- | --- |
| c1159_g1 | AT4G15530 | Carbohydrate metabolism | Encodes a dual-targeted protein believed to act as a pyruvate, orthophosphate dikinase. These enzymes are normally associated with C4 photosynthesis which does not occur in Arabidopsis. However, PPDK may play a role in remobilizing nitrogen during leaf senescence in Arabidopsis. The product of the long transcript (.1 gene model) was shown to be targeted to the chloroplast, whereas the shorter transcript (no targeting sequence) accumulates in the cytosol. The two proteins were also found to be expressed in slightly different tissues. |
| c32856_g1 | AT5G63840 | Carbohydrate metabolism | Encodes the alpha-subunit of a glucosidase II enzyme. A mutant has been shown to be specifically impaired in cellulose production. |
| c135343_g1 | AT5G67230 | Carbohydrate metabolism | Encodes a member of the GT43 family glycosyltransferases involved in glucuronoxylan biosynthesis: AT2G37090 (IRX9) and AT1G27600 (IRX9-L or I9H, IRX9 homolog); AT4G36890 (IRX14) and AT5G67230 (IRX14-L or I14H, IRX14 homolog). They form two functionally non-redundant groups essential for the normal elongation of glucuronoxylan backbone. I9H functions redundantly with IRX9, I14H is redundant with IRX14. IRX9 or I9H do not complement IRX14, IRX14 or I14H do not complement IRX9. |
| c51737_g1 | AT1G78240 | Carbohydrate metabolism | Encodes TSD2 (TUMOROUS SHOOT DEVELOPMENT2), a putative methyltransferase with an essential role in cell adhesion, anthocyanin accumulation, and coordinated plant development. |
| c134143_g1 | AT5G53370 | Carbohydrate metabolism | pectin methylesterase PCR fragment F;(source:Araport11) |
| c18089_g2 | AT2G45290 | Carbohydrate metabolism | Transketolase;(source:Araport11) |

Table S9-2 Candidates' genes of *Hornstedtia hainanensis* involved in mucilage-like pollen coat formation (continued)

| Unigene ID | Arabidopsis accession | Function | TAIR Description |
| --- | --- | --- | --- |
| c60350_g1 | AT5G56360 | Carbohydrate metabolism | Encodes PSL4, beta-subunit of endoplasmic reticulum-resident glucosidase II, which is essential for stable accumulation and quality control of the elf18 receptor EFR but not the flg22 receptor FLS2. The mRNA is cell-to-cell mobile. |
| c65065_g2 | AT5G55070 | Carbohydrate metabolism | Encodes the E2 subunit of the 2-oxoglutarate dehydrogenase. |
| c67376_g1 | AT4G34700 | Carbohydrate metabolism | Encodes the B22 subunit of eukaryotic mitochondrial Complex I. Mutation in the gene display pleiotropic phenotypes including shorter roots, smaller plants and delayed flowering. The mRNA is cell-to-cell mobile. |
| c59984_g1 | AT3G50520 | Carbohydrate metabolism | Phosphoglycerate mutase family protein;(source: Araport11) |
| c94654_g1 | AT1G06550 | Carbohydrate metabolism | ATP-dependent caseinolytic (Clp) protease/crotonase family protein;(source:Araport11) |
| c56779_g2 | AT5G41040 | Carbohydrate metabolic process / Lipid metabolism | Encodes a feruloyl-CoA transferase required for suberin synthesis. Has feruloyl-CoA-dependent feruloyl transferase activity towards substrates with a primary alcohol. |

Table S10 Expression pattern of some genes related to pollen wall formation in four gingers, *Caulokaempferia coenobialis* (Cco), *Hornstedtia hainanensis* (Hhn), *Pyrgophyllum yunnanense* (Pyn), *Zingiber nudicarpum* (Znu) and their homologs in *Arabidopsis thaliana* (At).

|  | | FPKM value | | | Known genes related to pollen wall formation | | |
| --- | --- | --- | --- | --- | --- | --- | --- |
| GENE ID | | MMC | TET | BCP | Gene name | Function | Reference |
| Cco | c45763_g1 | 66.59 | 2.29 | – | TDF1/MYB35 | tapetal differentiation and function | Feng et al., 2012 |
| Hhn | c32896_g1 | 213.15 | 185.31 | 12.90 |  |  |  |
| Pyn | c13032_g1 | 96.77 | 26.62 | 0.57 |  |  |  |
| Znu | Cluster-31632.115832 | 286.34 | 81.63 | 4.32 |  |  |  |
| At | AT3G28470 | 87.31 | 6.96 | 4.81 |  |  |  |
|  |  |  |  |  |  |  |  |
| Cco | c80107_g1 | 11.26 | 27.62 | 87.61 | AtUSP | intine development | Schnurr et al., 2006 |
| Hhn | c66714_g1 | 43.54 | 35.52 | 49.27 |  |  |  |
| Pyn | c17816_g1 | 41.11 | 65.39 | 121.49 |  |  |  |
| Znu | Cluster-31632.124343 | 41.86 | 39.50 | 120.72 |  |  |  |
| At | AT5G52560 | 172.23 | 211.66 | 234.35 |  |  |  |
|  |  |  |  |  |  |  |  |
| Cco | c64119_g11 | 14.44 | 9.66 | 9.98 | TKPR1 | sporopollenin monomer biosynthesis | Grienenberger et al. 2010 |
| Hhn | c55194_g1 | 70.53 | 177.56 | 283.54 |  |  |  |
| Pyn | c46014_g1 | 1.12 | 1.51 | 0.81 |  |  |  |
| Znu | Cluster-31632.171853 | 3.16 | 2.41 | 2.35 |  |  |  |
| At | AT4G35420 | 1296.33 | 293.20 | 28.41 |  |  |  |
|  |  |  |  |  |  |  |  |
| Cco | - | - | - | - | LAP3 | exine synthesis | Dobritsa et al. 2009 |
| Hhn | - | - | - | - |  |  |  |
| Pyn | - | – | - | - |  |  |  |
| Znu | - | - | - | - |  |  |  |
| At | AT3G59530 | 787.93 | 470.91 | 205.63 |  |  |  |
|  |  |  |  |  |  |  |  |
| Cco | - | - | - | - | LAP5 | exine synthesis | Dobritsa et al. 2010 |
| Hhn | - | - | - | - |  |  |  |
| Pyn | - | - | - | - |  |  |  |
| Znu | - | - | - | - |  |  |  |
| At | AT4G34850 | 1131.09 | 237.31 | 4.75 |  |  |  |
|  |  |  |  |  |  |  |  |
| Cco | - | - | - | - | LAP6 | exine synthesis | Dobritsa et al. 2010 |
| Hhn | - | - | - | - |  |  |  |
| Pyn | - | - | - | - |  |  |  |
| Znu | - | - | - | - |  |  |  |
| At | AT1G02050 | 1260.8 | 316.05 | 18.96 |  |  |  |
|  |  |  |  |  |  |  |  |
| Cco | - | - | - | - | ABCG26 | transfer the sporopollenin lipid precursors | Choi et al., 2011 |
| Hhn | - | - | - | - |  |  |  |
| Pyn | - | - | - | - |  |  |  |
| Znu | - | - | - | - |  |  |  |
| At | AT3G13220 | 513.05 | 166.96 | 2.68 |  |  |  |

“-”no expression was detected, MMC = Microspore mother cell stage, TET = Tetrad stage, and BCP = Bicellular pollen stage
